# Dynamic albumin-to–RDW trajectories for time-dependent risk stratification in acute brain injury

**DOI:** 10.3389/fnut.2026.1855636

**Published:** 2026-08-17

**Authors:** Juan Wang, Man-Man Xu, Zheng Peng, Wen-Juan Li, Meng-Lian Duan, Chun-Hua Hang, Peng-Lai Zhao

**Affiliations:** 1 Department of Neurosurgery, Nanjing Drum Tower Hospital, Affiliated Hospital of Medical School, Nanjing University, Nanjing, China; 2 Department of Neurosurgery, Nanjing Drum Tower Hospital Clinical College of Nanjing University of Chinese Medicine, Nanjing, China; 3 Neurosurgical Institute, Nanjing University, Nanjing, China; 4 Center for Medical Big Data, Nanjing Drum Tower Hospital, The Affiliated Nanjing Drum Tower Hospital of Nanjing University Medical School, Nanjing, China

**Keywords:** acute brain injury, albumin-to–RDW ratio (ARR), external validation, machine learning, standardized trajectories, time-stratified cox

## Abstract

**Background:**

Acute brain injury (ABI) requires early, interpretable risk stratification to support ICU risk assessment. We evaluated whether longitudinal albumin-to–red cell distribution width ratio (ARR) trajectories provide time-dependent prognostic information and support prediction with multicenter validation.

**Methods:**

In this multicenter, retrospective cohort study (NSICU, MIMIC-IV, eICU), latent class growth modeling identified ARR trajectories. Nonproportional hazards were addressed with time-stratified Cox models (0–7, 7–14, >14 days) and restricted mean survival time (RMST) at 7, 14, and 28 days. Multiple machine-learning classifiers were benchmarked to develop an interpretable prediction model with internal and external validation. Performance assessment included discrimination, calibration, decision-curve analysis, and SHAP-based interpretability.

**Results:**

Among 8,270 ICU patients (NSICU n = 5,093; MIMIC-IV n = 744; eICU n = 2,433), four ARR trajectories were identified. Relative to Gradual Decline, recovery-type trajectories were associated with lower early mortality risk (0–7 days: HRs 0.29 and 0.14; 7–14 days: HRs 0.43 and 0.50), whereas the Sustained High-Risk trajectory showed excess hazard primarily beyond 14 days (HR 1.42). These time-dependent patterns were broadly supported by RMST and sensitivity analyses. The final ExtraTrees model achieved AUROC 0.869 and Brier score 0.066 on the internal hold-out set; external validation yielded AUROC 0.731, PR AUC 0.429, and Brier score 0.161, indicating attenuated but informative external performance with modest decision-curve benefit. A web-based research calculator provides individualized risk estimates and SHAP explanations.

**Conclusion:**

Standardized ARR trajectories provide dynamic, phase-specific prognostic information in ABI and complement static assessments. When integrated into an interpretable machine-learning model, these trajectories may support adjunctive risk estimation as patient status evolves. Prospective evaluation and site-level recalibration are warranted.

## Graphical abstract

**Figure:**
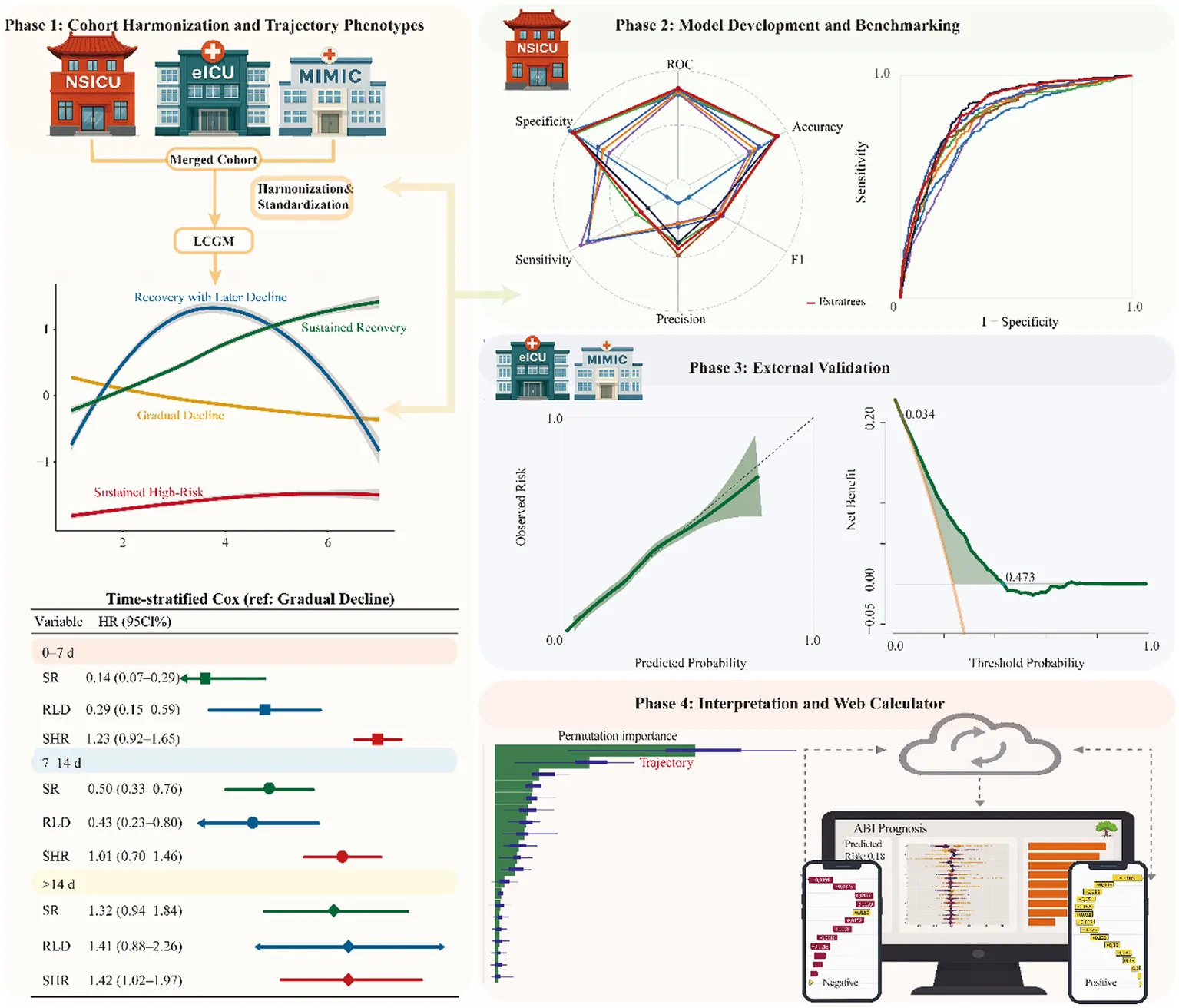
The graphical abstract summarizes four phases: (1) cohort harmonization and LCGM-derived ARR trajectories (SHR, GD, RLD, SR) with time-stratified Cox models using Gradual Decline as the reference; (2) model development and cross-validated benchmarking in the NSICU training cohort, leading to selection of ExtraTrees; (3) external validation with calibration assessment and decision-curve analysis; and (4) SHAP-based model interpretation and implementation as a web-based research calculator for individualized risk estimation and model explanation.

## Introduction

Acute brain injury (ABI), encompassing traumatic brain injury (TBI) and stroke, requires early, actionable risk stratification to inform ICU and perioperative management, including triage decisions. Contemporary guidance emphasizes organized regional systems, timely triage, and access to neurocritical care and neurosurgical capabilities across the prehospital-to-postacute continuum (1, 2). Yet adherence to process measures alone may be insufficient. In a multicenter trauma consortium, hospital-level compliance with selected Brain Trauma Foundation processes—intracranial pressure (ICP) monitoring and craniotomy—showed no clear association with risk-adjusted mortality in severe TBI (3). For surgeons and intensivists, these observations underscore the need for patient-level, outcome-oriented risk stratification that may help inform phase-specific care rather than rely solely on checklist compliance.

Current ABI risk stratification largely relies on static clinical scores and single-time-point laboratory markers. Established scales such as CRASH distill baseline severity but are not designed to track evolving physiology or support phase-specific ICU and perioperative care decisions (4). In parallel, surgical and emergency settings increasingly use readily obtainable hematologic indices and simple composite ratios, yet their prognostic application typically remains cross-sectional (5, 6). Ward-level initiatives aimed at early identification of high-risk surgical patients further underscore a system-level focus on processes rather than patient-level dynamic risk (7). Even within neurotrauma, emerging composite markers illustrate interest in accessible signals without addressing longitudinal behavior or time-dependent risk (8). Collectively, these prevailing approaches seldom standardize biomarker trajectories across centers or formally model time-varying risk, constraining transportability and clinical interpretation.

Accordingly, we aimed to characterize longitudinal albumin-to–red cell distribution width ratio (ARR) patterns in multicenter ABI ICU cohorts and assess their time-dependent associations with in-hospital mortality using time-stratified Cox models and restricted mean survival time (RMST). Additionally, we evaluated whether ARR trajectory information could be integrated into an interpretable prediction framework with internal and external validation.

## Methods

### Data sources

We conducted a multicenter, retrospective cohort study of adults with ABI using one institutional neurosurgical ICU (NSICU, 2018–2024) and two public ICU databases (MIMIC-IV v3.1, 2008–2022; eICU v2.0, 2014–2015). NSICU supported model development and internal validation, whereas MIMIC-IV and eICU served as independent external validation cohorts. Reporting followed STROBE and TRIPOD guidance for observational prediction-model studies with external validation. Ethical approvals and consent procedures are detailed under Ethics (9, 10).

### Data collection

MIMIC-IV and eICU were queried via structured SQL; NSICU data were captured with an electronic case-report form workflow. To ensure cross-cohort comparability, we harmonized variable names, units, categorical encodings, and measurement windows; screened physiologically implausible values *a priori*; and assembled longitudinal series for ARR. Key variable domains (demographics, comorbidities, admission laboratories, interventions, outcomes) are summarized in Supplementary Table 1, and variable-level missingness is summarized in Supplementary Table 2 (with imputation diagnostics shown in Supplementary Figure 2). The patient-selection and study workflow is shown in Supplementary Figure 1.

### Study population

The target population comprised adults with ABI admitted to an ICU. To avoid duplication, we retained only the index ICU admission for the first hospitalization per patient. Prespecified exclusions were age <18 years, ICU length of stay <24 h, missing in-hospital mortality status, and <3 paired albumin–RDW measurements for trajectory modeling. The primary outcome was in-hospital mortality, defined from ICU admission to hospital discharge or death; survivors were censored at discharge. Discharge disposition and ICU/hospital lengths of stay were recorded as secondary descriptive measures.

### ARR construction and trajectory modeling

Albumin (g/L) and RDW (%) were unit-harmonized across sources and paired at each time point to compute ARR (albumin/RDW). Serial measurements during the early ICU stay were aligned to prespecified daily sequential windows (0–168 h after ICU admission) anchored at ICU admission to select at most one paired albumin–RDW value per window and limit bias from dense sampling. When multiple values occurred within a window, we selected the observation closest to the window midpoint (ties resolved by the earlier value). Windows without an available paired albumin–RDW observation were recorded as missing; ARR values were not imputed. For quality control, physiologically implausible values were flagged as missing, and extreme yet plausible observations were winsorized at the 0.5th–99.7th percentiles to limit undue influence. To mitigate inter-laboratory variability prior to trajectory learning, cleaned ARR values were standardized to z scores within each database (mean 0, SD 1); raw ARR was retained for descriptive plots. Sensitivity analyses evaluated an expanded ≥2-pair cohort and an alternative 10-window trajectory construction.

Latent class growth modeling (LCGM) was applied to standardized ARR to estimate candidate 2–6-class solutions. Model selection considered AIC, BIC, sample-size–adjusted BIC (SABIC), entropy, average posterior probabilities (APPs), minimum class size, and clinical interpretability (11). Each patient was assigned to the most likely class by maximum posterior probability; APPs and high-confidence posterior-probability sensitivity analyses were reported to evaluate classification uncertainty.

### Time-to-event analyses

Survival was summarized with Kaplan–Meier estimates and compared using the log-rank test. We first fit a Cox proportional-hazards (PH) model with trajectory indicators (reference: Gradual Decline) and evaluated the PH assumption using graphical diagnostics and Schoenfeld residual tests. Because PH was violated, we implemented time-stratified Cox models with prespecified intervals of 0–7, 7–14, and >14 days to reflect early, intermediate, and late ICU phases, reporting hazard ratios with 95% CIs. Covariates were selected *a priori* based on clinical relevance and prior literature, with additional inclusion via a ≥ 10% change-in-estimate criterion: Model 1 unadjusted; Model 2 adjusted for age, ICU type, TBI, temperature, heart rate, RBC, platelets, BUN, glucose, sodium, CKD, and CCI; Model 3 additionally adjusted for SOFA, admission GCS, mechanical ventilation, embolization, craniotomy, and vasopressor use. As a PH-robust complement, we estimated restricted mean survival time (RMST) differences at *τ* = 7, 14, and 28 days using established procedures.

### Model development and validation

After deriving ARR trajectories, the trajectory class was included as a categorical predictor alongside prespecified clinical variables (domains in Supplementary Table 1). To prevent data leakage, multiple imputation by chained equations was performed within each cross-validation (CV) training fold; categorical predictors, including the derived ARR trajectory class, were encoded within the modeling pipeline.

We benchmarked 14 classifiers using stratified 10-fold CV in the NSICU training cohort: XGBoost, support vector machine (SVM), classification and regression trees (CART), random forest, neural network (multilayer perceptron), Naive Bayes, logistic regression, LightGBM, k-nearest neighbors (k-NN), gradient boosting machine (GBM), extremely randomized trees (ExtraTrees), CatBoost, Bayesian Network (BayesNet), and AdaBoost. Within each fold, preprocessing, imputation, Boruta feature selection, model fitting, and hyperparameter tuning (grid/random search) were executed inside the resampling loop. Primary metrics were AUROC, PR AUC, and Brier score; we also reported accuracy, sensitivity, specificity, positive predictive value (PPV), and F1.

The NSICU cohort was split by stratified sampling into a training subset (67%)—used for CV and tuning—and an internal hold-out set (33%) for confirmatory assessment under the same metric framework. For external validation, the locked model—comprising the selected feature set and tuned hyperparameters—was applied without refitting to MIMIC-IV and eICU. A CV-derived operating threshold selected by maximizing the F1 score in the training cohort was held fixed for the internal hold-out and external evaluations. Calibration was assessed using calibration intercept/slope and calibration plots against the 45° reference line. Clinical utility was examined with decision-curve analysis (DCA). Model interpretability was assessed with SHAP and permutation−/loss-based importance (DALEX, IML) to elucidate global and case-level drivers of risk.

### Statistical analysis

Normality of continuous variables was assessed with the Kolmogorov–Smirnov test. Data were summarized as mean (SD) or median (IQR), as appropriate; categorical variables as n (%). Two-group comparisons used the independent t test or Mann–Whitney U test for continuous data and the chi-square or Fisher’s exact test for categorical data. Across ARR trajectory classes, group differences were examined with one-way ANOVA (or Kruskal–Wallis when assumptions were not met), followed by Tukey’s HSD (after ANOVA) or Dunn’s test with multiplicity adjustment (after Kruskal–Wallis) for post-hoc contrasts. Statistical significance was defined as two-sided *p* < 0.05. LCGM was fitted using the lcmm package in R; all analyses were performed in R (version 4.2.2) and the Free Statistics analysis platform.

## Results

The final study cohort included 8,270 ICU patients from three databases: NSICU (*n* = 5,093), MIMIC-IV (*n* = 744), and eICU (*n* = 2,433). The NSICU database was designated for model training and internal validation, while the MIMIC-IV and eICU databases served as independent external validation cohorts. Data were harmonized across databases to ensure cross-cohort comparability (Supplementary Figure 1).

### ARR trajectory identification and baseline characteristics

**Figure 1:**
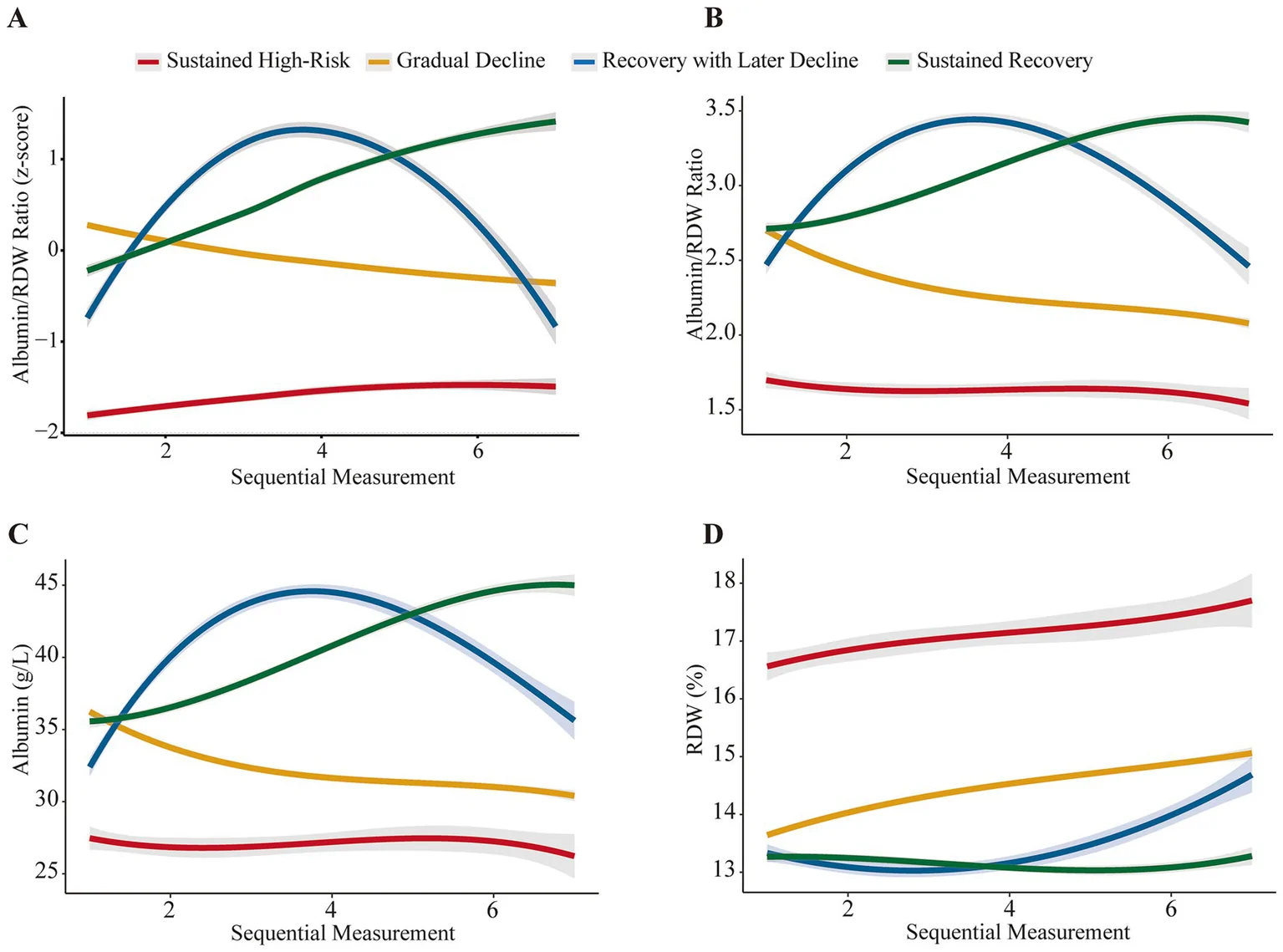
Four distinct ARR trajectory phenotypes. Panels **(A–D)** show the LCGM-derived ARR trajectory phenotypes across sequential paired measurements for **(A)** standardized ARR (z-score), **(B)** ARR, **(C)** albumin (g/L), and **(D)** RDW (%). Solid lines indicate model-estimated class means and shaded bands denote 95% CIs. Colors denote phenotypes: Sustained High-Risk (red), Gradual Decline (orange), Recovery with Later Decline (blue), and Sustained Recovery (green).

Based on the overall balance of model-fit indices, classification certainty, class size, trajectory separation, and clinical interpretability, the four-class LCGM solution was selected for the primary analysis (Supplementary Table 3). The four trajectories were designated as Sustained High-Risk (SHR, persistently low ARR with low albumin and rising RDW), Gradual Decline (GD, gradual ARR decrease with slight albumin decline and RDW increase), Recovery with Later Decline (RLD, early ARR improvement followed by late decline with intermittent RDW increases), and Sustained Recovery (SR, steady ARR increase with rising albumin and relatively stable RDW) (Figure 1). Sensitivity analyses in the expanded ≥2-pair cohort and the 10-window trajectory construction reproduced the major trajectory patterns, whereas separately modeled albumin and RDW trajectories supported the biological interpretation of ARR as a composite dynamic marker (Supplementary Tables 4–7; Supplementary Figures 3–5).

Baseline characteristics differed across trajectories (Table 1; Supplementary Table 8). SHR was characterized by greater systemic illness burden and treatment intensity, including higher SOFA scores, lower RBC and platelet counts, higher BUN, more frequent CKD, mechanical ventilation, vasopressor use, and craniotomy. GD generally reflected a lower-acuity profile. RLD and SR showed early recovery-type ARR patterns but had higher proportions of neurological ICU admission and neurosurgical interventions, suggesting that trajectory membership was related to both baseline severity and management context. Quantitative profiles and discharge disposition across trajectories are shown in Supplementary Figure 6. Baseline characteristics of patients with two versus ≥3 paired measurements are shown in Supplementary Table 9.

**Table 1:** Baseline characteristics across ARR trajectory classes.

Characteristics	Total	Sustained High-Risk	Gradual Decline	Recovery with Later Decline	Sustained Recovery	*p*
	(*n* = 8,270)	(*n* = 444)	(*n* = 6,803)	(*n* = 351)	(*n* = 672)	
Age, years	58.9 ± 15.4	59.4 ± 14.4	59.2 ± 15.5	56.0 ± 14.2	56.6 ± 14.2	<0.001
ICU type	5,729 (69.3)	310 (69.8)	4,510 (66.3)	324 (92.3)	585 (87.1)	<0.001
TBI	2,136 (25.8)	105 (23.6)	1742 (25.6)	127 (36.2)	162 (24.1)	<0.001
Temperature, °C	36.6 ± 0.8	36.5 ± 1.0	36.6 ± 0.8	36.6 ± 0.8	36.6 ± 0.7	0.202
Heart rate, bpm	88.0 ± 24.3	92.8 ± 26.2	88.3 ± 24.8	83.3 ± 16.5	84.1 ± 20.3	<0.001
RBC, ×10^12^/L	3.8 ± 0.8	3.3 ± 0.9	3.9 ± 0.8	3.5 ± 0.7	3.9 ± 0.7	<0.001
Platelet, ×10^9^/L	185.0 ± 79.4	167.0 ± 112.3	188.1 ± 77.7	162.0 ± 67.1	177.5 ± 72.3	<0.001
BUN, mg/dL	14.5 (10.9, 20.2)	18.0 (12.3, 29.1)	14.3 (10.9, 20.0)	13.2 (10.1, 19.3)	14.3 (11.0, 19.5)	<0.001
Glucose, mg/dL	138.1 ± 67.2	145.3 ± 68.0	137.4 ± 68.1	134.7 ± 51.3	142.4 ± 64.3	0.022
Sodium, mmol/L	140.4 ± 5.4	141.3 ± 7.8	140.2 ± 5.2	141.2 ± 6.0	140.9 ± 4.8	<0.001
CKD	1,218 (14.7)	117 (26.4)	858 (12.6)	91 (25.9)	152 (22.6)	<0.001
CCI	3.0 (2.0, 5.0)	4.0 (2.0, 6.0)	3.0 (2.0, 5.0)	3.0 (2.0, 5.0)	3.0 (2.0, 5.0)	<0.001
SOFA	4.0 (2.0, 6.0)	6.0 (4.0, 9.0)	4.0 (2.0, 6.0)	5.0 (4.0, 7.0)	4.0 (3.0, 6.0)	<0.001
GCS at admission	10 ± 5	9 ± 5	10 ± 5	7 ± 5	7 ± 5	<0.001
Ventilation	3,512 (42.5)	229 (51.6)	2,638 (38.8)	207 (59.1)	438 (65.3)	<0.001
Embolization	2,755 (33.3)	98 (22.1)	2,350 (34.5)	95 (27.1)	212 (31.5)	<0.001
Craniotomy	2,578 (31.2)	173 (39)	1815 (26.7)	206 (58.7)	384 (57.1)	<0.001
Vasopressor	1,250 (15.1)	134 (30.2)	983 (14.4)	48 (13.7)	85 (12.6)	<0.001

Continuous variables are summarized as mean ± SD or median (IQR), as appropriate; categorical variables as *n* (%). ARR trajectory classes were defined as Sustained High-Risk, Gradual Decline, Recovery with Later Decline, and Sustained Recovery. ARR, albumin-to–red cell distribution width ratio; RBC, red blood cell count; BUN, blood urea nitrogen; CKD, chronic kidney disease; CCI, Charlson comorbidity index; SOFA, Sequential Organ Failure Assessment; GCS, Glasgow Coma Scale.

### ARR trajectories and prognosis

**Figure 2:**
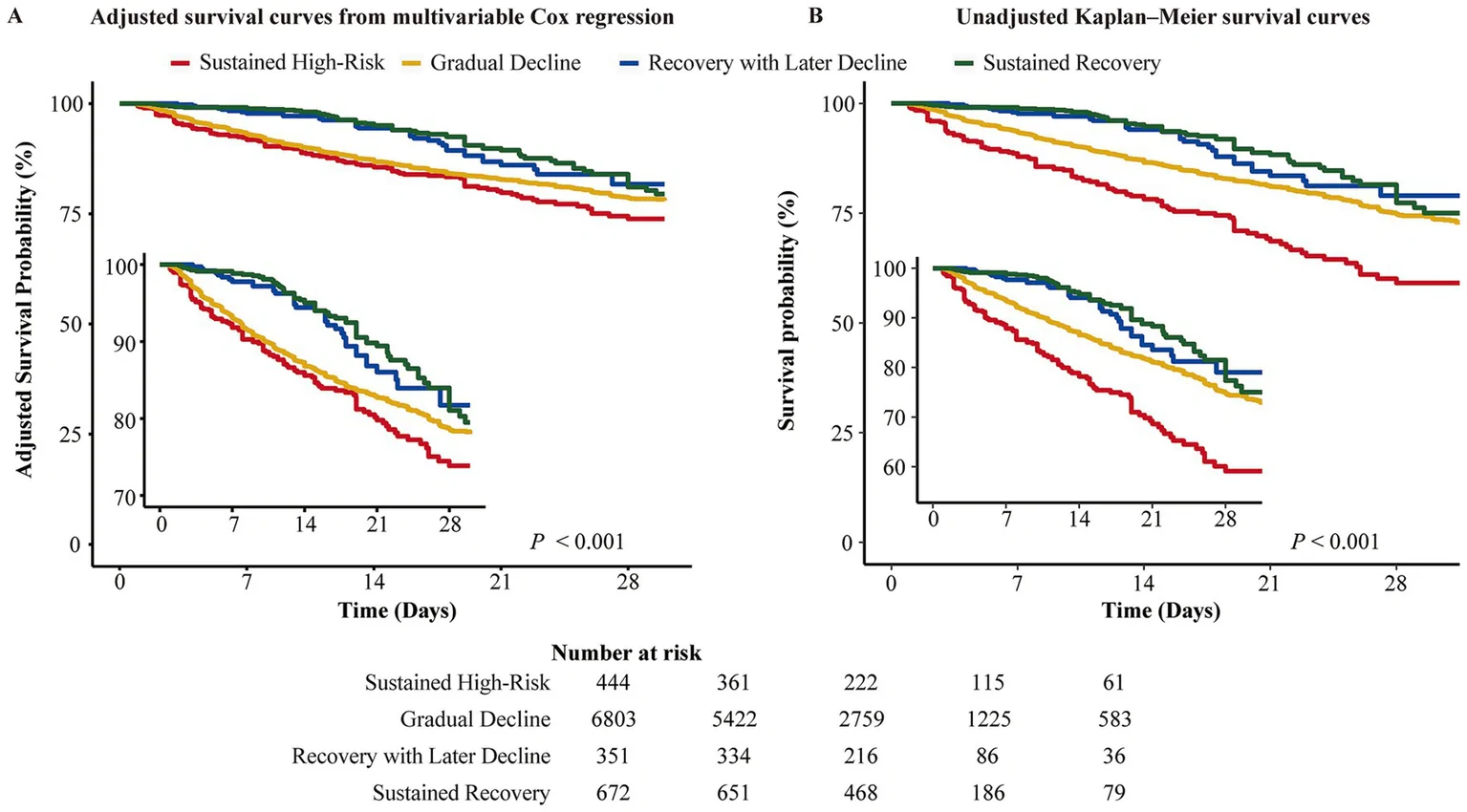
28-day survival by ARR trajectory class. Panels **(A,B)** show 28-day survival across the four ARR trajectory phenotypes (Sustained High-Risk, Gradual Decline, Recovery with Later Decline, and Sustained Recovery). Colors are red, orange, blue, and green, respectively. **(A)** Covariate-adjusted survival curves estimated from the multivariable Cox model adjusted for age, ICU type, TBI, temperature, heart rate, RBC, platelet, BUN, glucose, sodium, CKD, CCI, SOFA, GCS at admission, ventilation, embolization, craniotomy, and vasopressor use. **(B)** Unadjusted Kaplan–Meier curves.

Survival differed significantly across trajectories (*p* < 0.001; Figure 2), with SHR showing the least favorable survival pattern. Because the survival curves crossed, indicating nonproportional hazards (*p* < 0.001 for the initial Cox model; Supplementary Figure 7A), we fitted time-stratified Cox models, which showed improved PH behavior with no evidence of violation (Supplementary Figure 7B). During 0–7 days, SHR did not differ significantly from GD (HR 1.23, 95% CI 0.92–1.65; *p* = 0.159; Table 2). RMST analyses (Model 3, fully adjusted) showed small differences between GD and SHR at 7 days (+0.08 days; *p* = 0.191), with larger differences at 14 days (+0.38 days; *p* = 0.025) and 28 days (+1.77 days; *p* < 0.001) (Supplementary Table 11; Supplementary Figure 8). At 0–7 days, versus GD, RLD was associated with lower mortality risk (HR 0.29, 95% CI 0.15–0.59; p < 0.001), as was SR (HR 0.14, 95% CI 0.07–0.29; *p* < 0.001). At 7–14 days, RLD (HR 0.43, 95% CI 0.23–0.80; *p* = 0.008) and SR (HR 0.50, 95% CI 0.33–0.76; *p* = 0.001) remained associated with lower mortality risk. Beyond 14 days, SHR showed higher risk versus GD (HR 1.42, 95% CI 1.02–1.97; *p* = 0.038), whereas RLD (HR 1.41, 95% CI 0.88–2.26; *p* = 0.155) and SR (HR 1.32, 95% CI 0.94–1.84; *p* = 0.107) were not significantly different from GD. Sensitivity analyses using alternative 5-day cutpoints and high-confidence trajectory assignments showed similar overall patterns (Supplementary Table 10; Supplementary Figure 9).

**Table 2:** Time-stratified cox models of in-hospital mortality by ARR trajectory phenotype.

Time interval	Model 1		Model 2		Model 3	
	HR (95% CI)	*p*	HR (95% CI)	*p*	HR (95% CI)	*p*
0–7 days						
Gradual Decline	Ref		Ref		Ref	
Sustained High-Risk	1.93 (1.45–2.57)	<0.001	1.47 (1.10–1.98)	0.009	1.23 (0.92–1.65)	0.159
Recovery with Later Decline	0.34 (0.17–0.69)	0.003	0.38 (0.19–0.77)	0.007	0.29 (0.15–0.59)	<0.001
Sustained Recovery	0.16 (0.07–0.33)	<0.001	0.18 (0.08–0.37)	<0.001	0.14 (0.07–0.29)	<0.001
7–14 days						
Gradual Decline	Ref		Ref		Ref	
Sustained High-Risk	1.52 (1.06–2.18)	0.022	1.20 (0.83–1.73)	0.327	1.01 (0.70–1.46)	0.939
Recovery with Later Decline	0.46 (0.24–0.86)	0.015	0.53 (0.28–0.99)	0.048	0.43 (0.23–0.80)	0.008
Sustained Recovery	0.54 (0.36–0.82)	0.004	0.63 (0.41–0.96)	0.030	0.50 (0.33–0.76)	0.001
>14 days						
Gradual Decline	Ref		Ref		Ref	
Sustained High-Risk	1.71 (1.24–2.37)	0.001	1.46 (1.05–2.03)	0.024	1.42 (1.02–1.97)	0.038
Recovery with Later Decline	1.19 (0.75–1.90)	0.464	1.59 (0.99–2.54)	0.056	1.41 (0.88–2.26)	0.155
Sustained Recovery	1.20 (0.86–1.67)	0.280	1.51 (1.08–2.11)	0.015	1.32 (0.94–1.84)	0.107

Cox models were stratified by in-hospital time intervals (0–7, 7–14, and >14 days). ARR trajectory phenotype was the exposure, with Gradual Decline as the reference. Entries are HRs with 95% CIs and two-sided P values. Model 1 unadjusted. Model 2 adjusted for age, ICU type, TBI, temperature, heart rate, RBC, platelet, BUN, glucose, sodium, CKD, and CCI. Model 3 further adjusted for SOFA, GCS at admission, ventilation, embolization, craniotomy, and vasopressor use.

### Time-stratified subgroup analysis

**Figure 3:**
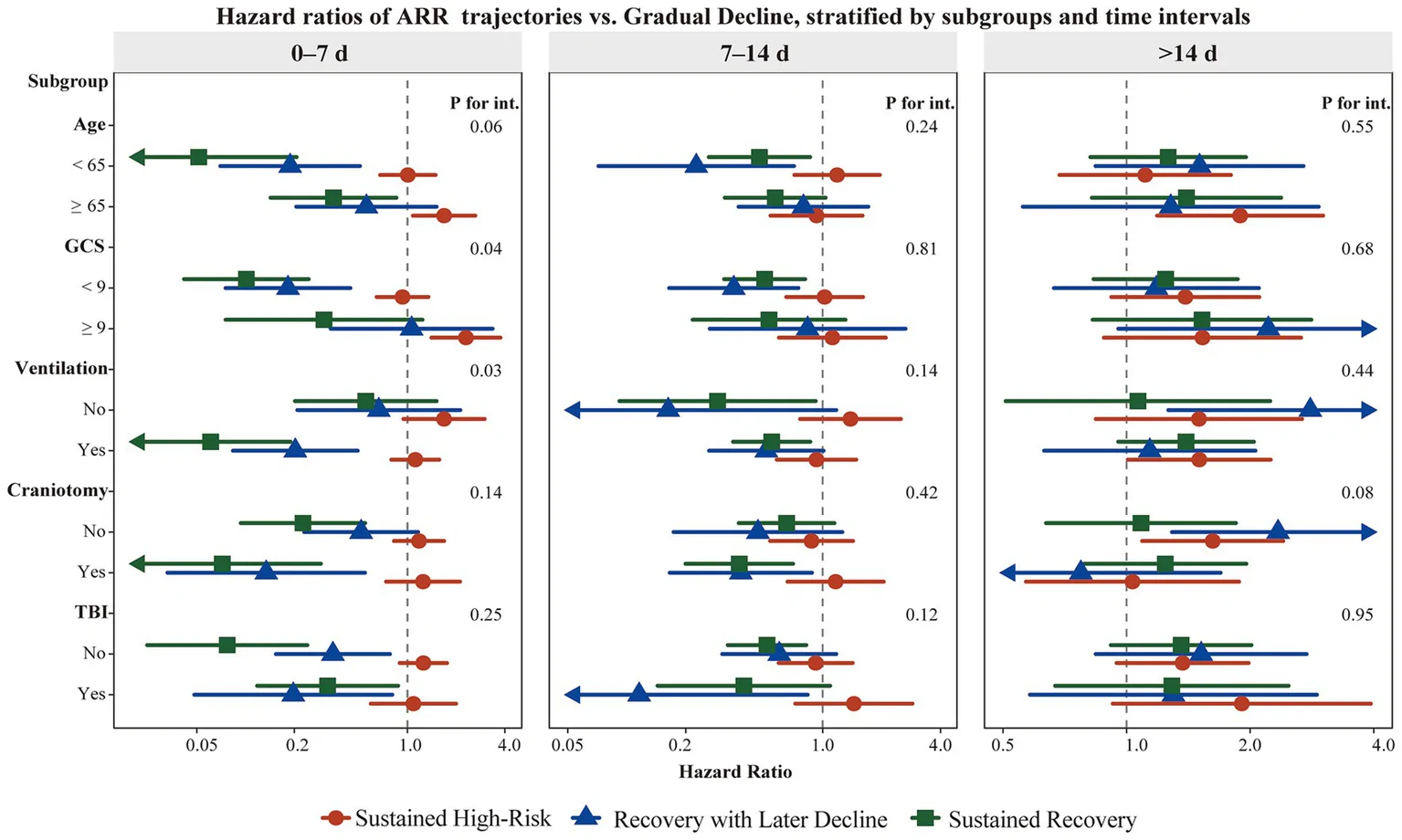
Subgroup hazard ratios by ARR trajectory stratified by time interval. Forest plots show HRs (95% CIs) from time-stratified multivariable Cox models comparing ARR trajectories with Gradual Decline (reference) across 0–7, 7–14, and >14 days and prespecified subgroups. Points indicate HRs and bars 95% CIs; the vertical dashed line denotes HR = 1. Colors/symbols indicate phenotypes: Sustained High-Risk (red circles), Recovery with Later Decline (blue triangles), and Sustained Recovery (green squares). “*P* for int.” is the likelihood-ratio test for the trajectory × subgroup interaction within each interval.

The time-stratified subgroup analysis (Figure 3) revealed dynamic associations with in-hospital mortality. During 0–7 days, RLD and SR were generally associated with lower mortality risk than GD across subgroups, with nominal interaction signals for admission GCS (*p* = 0.04) and mechanical ventilation (*p* = 0.03), which were exploratory and should be interpreted cautiously given multiple subgroup comparisons. Between 7 and 14 days, effect sizes attenuated and interaction tests were generally non-significant. After 14 days, subgroup-specific estimates for RLD and SR were not statistically significant, supporting a cautious interpretation of late-phase recovery-type trajectories. Additional subgroup results are shown in Supplementary Figure 10.

### Model development and benchmarking

**Figure 4:**
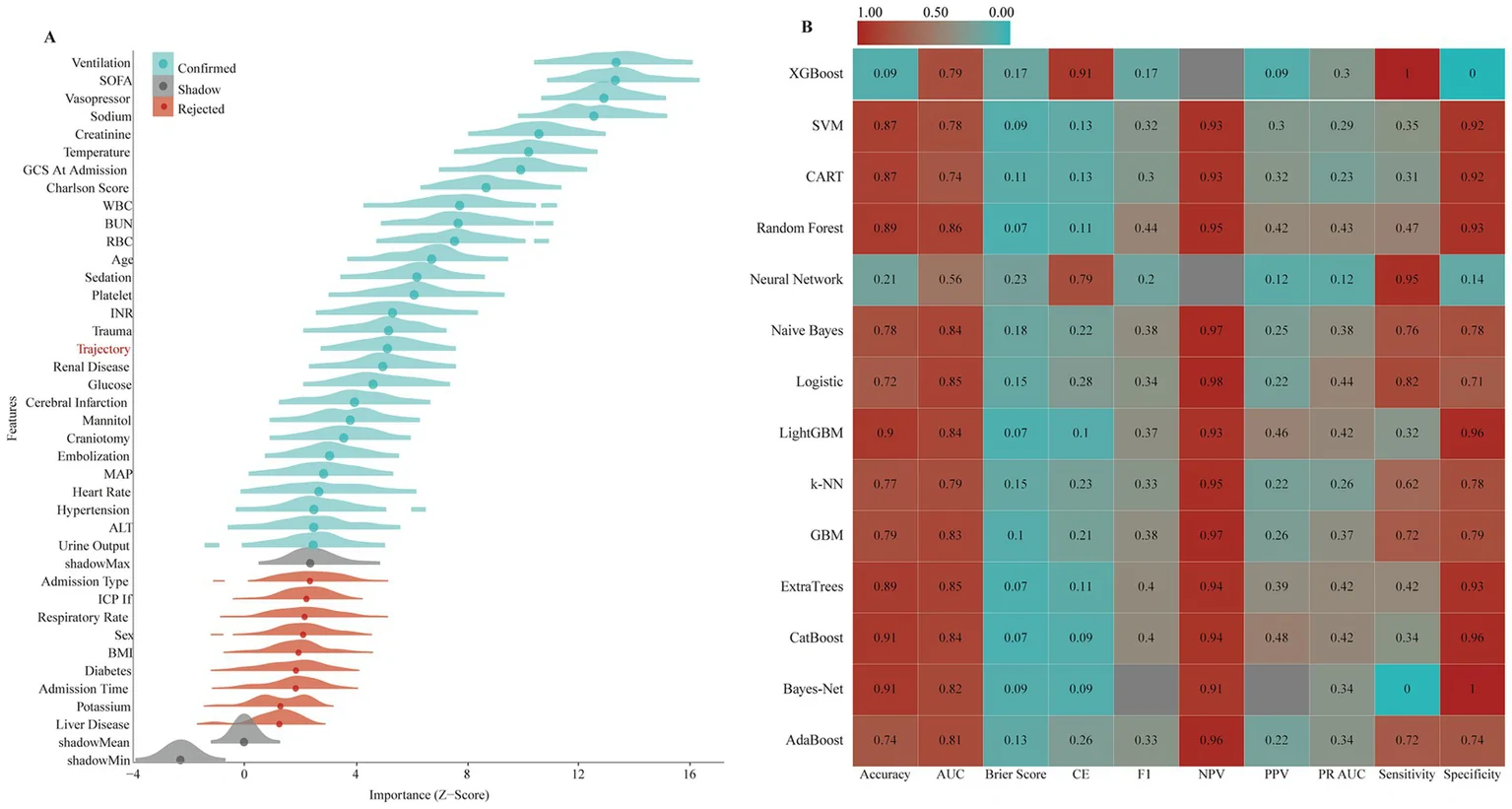
Boruta feature selection and cross-validated benchmarking of candidate classifiers. **(A)** Boruta feature-selection results based on importance Z-scores; features were classified as confirmed, shadow, or rejected. The ARR trajectory indicator (“Trajectory”) was confirmed and highlighted. **(B)** Heatmap of mean stratified 10-fold CV metrics for 14 classifiers. Grey cells indicate non-estimable metrics. Higher values indicate better performance except for Brier score and classification error.

**Figure 5:**
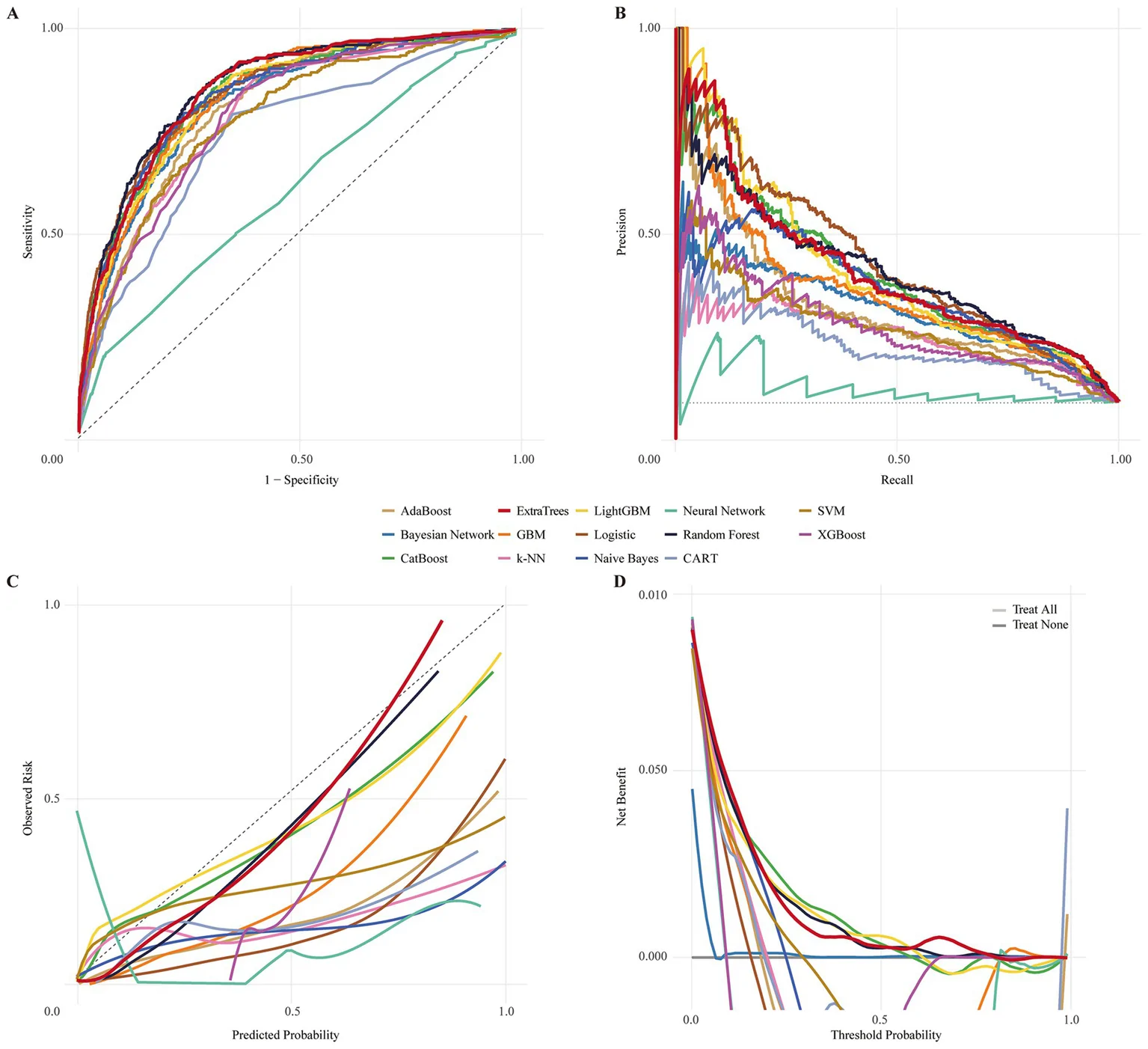
Cross-validated performance of benchmark classifiers. Stratified 10-fold CV performance of benchmark classifiers in the training cohort. **(A)** ROC curves. **(B)** Precision–recall curves; The horizontal dotted line indicates event prevalence. **(C)** LOESS-smoothed calibration curves with the 45° reference line. **(D)** Decision-curve analysis with treat-all and treat-none reference strategies.

Baseline characteristics differed between the NSICU derivation cohort and the external cohorts (MIMIC-IV/eICU) (Supplementary Table 12). After ARR-trajectory construction, Boruta retained a broad predictor set and confirmed ARR trajectory as a high-importance feature (Figure 4A). Fourteen candidate classifiers were benchmarked in the NSICU training cohort using stratified 10-fold CV across discrimination, calibration, and clinical-utility metrics (Figures 4B, 5; Supplementary Table 13). Based on the overall performance profile, ExtraTrees was selected for final model development.

**Figure 6:**
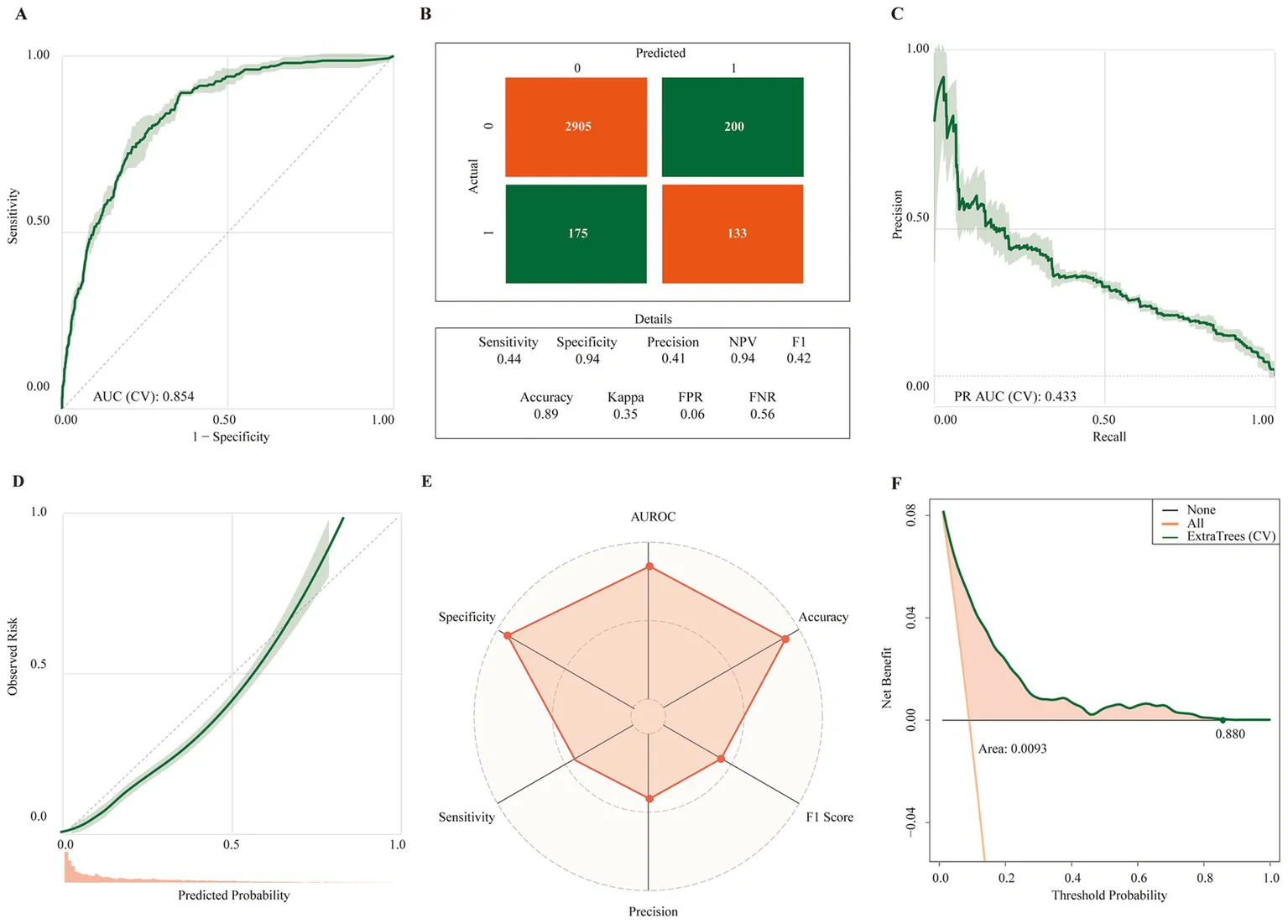
Cross-validated performance of the ExtraTrees classifier. ExtraTrees performance in the training cohort under stratified 10-fold cross-validation. **(A)** ROC curve with cross-validated AUC. **(B)** Confusion matrix at the CV-derived operating threshold with accuracy, sensitivity, specificity, precision, F1 score, and Cohen’s kappa. **(C)** Precision–recall curve with PR AUC. **(D)** Calibration plot (LOESS-smoothed observed vs. predicted risk) with the 45° reference line and a histogram of predicted probabilities. **(E)** Radar plot summarizing key metrics. **(F)** Decision-curve analysis with treat-none and treat-all reference strategies.

In CV, ExtraTrees achieved AUROC 0.854, PR AUC 0.433, Brier score 0.068, accuracy 0.890, sensitivity 0.435, specificity 0.936, PPV 0.407, and F1 score 0.416 (Figures 6A–F; Table 3; Supplementary Table 13). On the internal hold-out set, performance was similar, with AUROC 0.869, PR AUC 0.423, Brier score 0.066, accuracy 0.897, sensitivity 0.437, specificity 0.942, PPV 0.429, and F1 score 0.433 (Supplementary Figure 11; Supplementary Table 14). Pooled external validation showed attenuated but informative performance, with AUROC 0.731, PR AUC 0.429, Brier score 0.161, accuracy 0.703, sensitivity 0.557, specificity 0.748, PPV 0.406, and F1 score 0.470 (Supplementary Figure 12; Supplementary Table 15). DCA showed positive but modest net benefit across threshold probabilities of approximately 3.4–47.3% in external validation, compared with 1.0–88.0% in cross-validation and 1.0–54.5% in the internal hold-out set. Overall, ExtraTrees offered the most favorable trade-off across discrimination, calibration, PR AUC, and clinical-utility metrics (Figures 4–6; Supplementary Tables 13–15).

**Table 3:** Performance of the final tuned ExtraTrees classifier across development and validation datasets.

Cohort	AUROC	Accuracy	Brier score	F1 score	CE	PR AUC	Sensitivity	Specificity	NPV	PPV
CV	0.854	0.890	0.068	0.416	0.110	0.433	0.435	0.936	0.943	0.407
Training	1.000	1.000	0.000	1.000	0.000	1.000	1.000	1.000	1.000	1.000
Internal test	0.869	0.897	0.066	0.433	0.103	0.423	0.437	0.942	0.944	0.429
External validation	0.731	0.703	0.161	0.470	0.297	0.429	0.557	0.748	0.845	0.406

Values are reported for the final tuned ExtraTrees classifier. CV metrics are out-of-fold means from stratified 10-fold cross-validation. Training-set metrics are shown only as apparent performance and were not used for model selection. Internal and external metrics were calculated using the prespecified CV-derived F1-optimized threshold of 0.35.

### Model interpretation and web deployment

**Figure 7:**
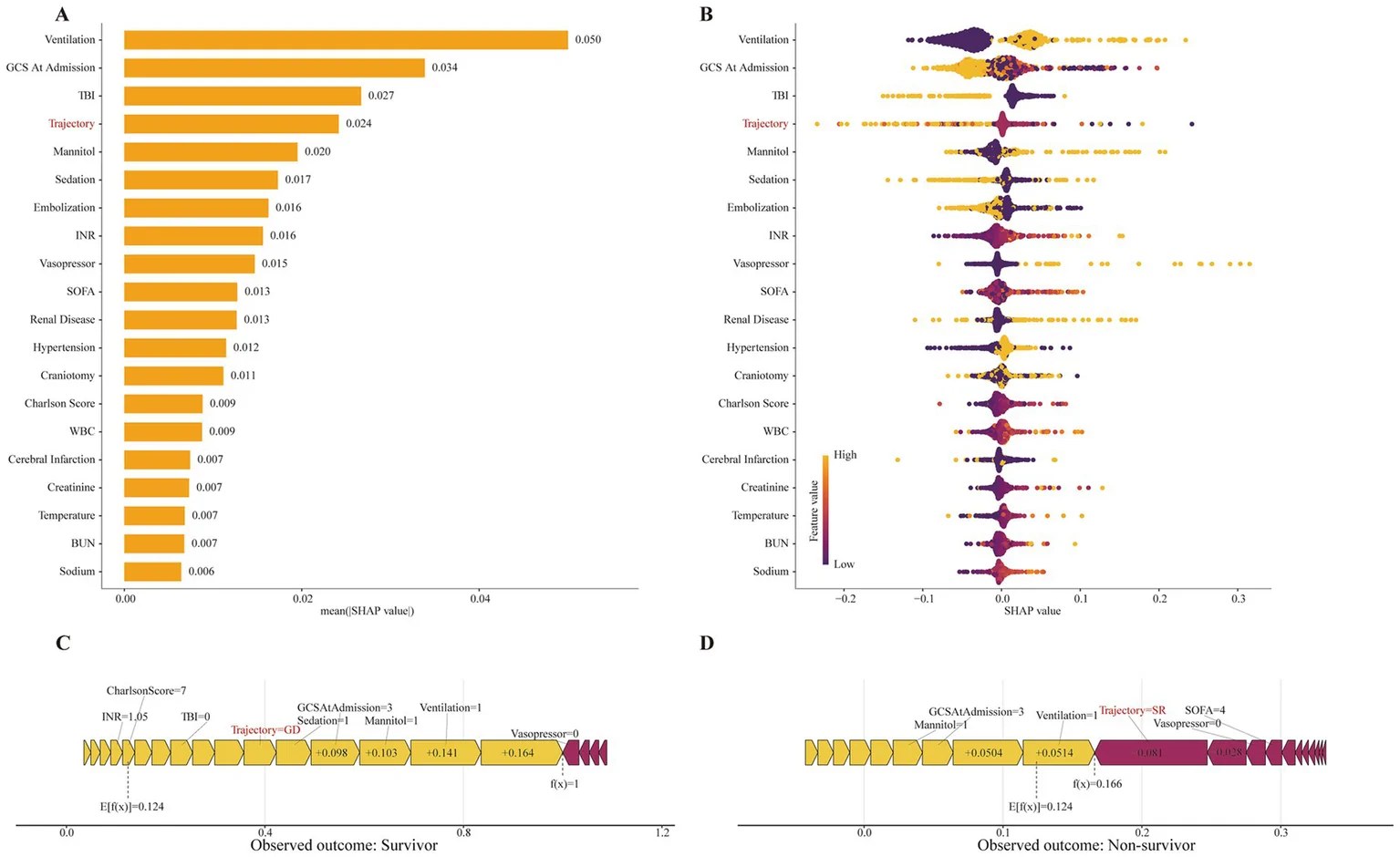
SHAP explanations for the ExtraTrees classifier in the training set. SHAP explanations computed in the training set **(A)** Global importance (mean |SHAP|) **(B)** SHAP summary (beeswarm): each dot represents a patient and SHAP values indicate each feature’s contribution to predicted risk (positive increases risk, negative decreases) **(C,D)** Representative force plots showing how features shift the prediction from the base value to the individual prediction; only the largest contributors are shown, with remaining features aggregated.

Global interpretation of the final ExtraTrees model highlighted mechanical ventilation, ARR trajectory, admission GCS, and TBI status as influential predictors (Figure 7A), with broadly concordant findings from DALEX and IML analyses (Supplementary Figure 13). The SHAP summary plot showed positive contributions for ventilation = 1 and lower GCS, with distinguishable effects across ARR trajectory classes; case-level force plots quantified individual predictions for representative high- and low-risk patients (Figures 7B–D). SHAP dependence plots further illustrated monotonic or stepwise shifts in predicted risk for key predictors (Supplementary Figures 14–15).

The final tuned model was implemented as a web-based research calculator that returns predicted in-hospital mortality and patient-specific SHAP explanations based on user-entered model variables (Supplementary Figure 16). The deployed calculator is available at https://njudrumtowernsicu.shinyapps.io/ABI_Prognosis_Model/. No user data are stored by the application. The calculator is provided for risk estimation and model explanation, pending prospective evaluation and local recalibration.

## Discussion

This multicenter cohort study of 8,270 ICU patients with ABI identified four distinct, standardized longitudinal ARR trajectories. Trajectory membership showed time-dependent associations with in-hospital mortality: recovery-type patterns were associated with lower early mortality risk (0–7 days HRs 0.29 and 0.14; 7–14 days HRs 0.43 and 0.50), whereas the SHR pattern carried excess hazard primarily beyond 14 days (HR 1.42). Exploratory subgroup analyses suggested stronger early associations in patients with greater baseline severity. Building on these signals, we developed an interpretable ExtraTrees model integrating ARR trajectories, with favorable internal performance and attenuated external validation performance, and implemented it as a web-based research calculator for individualized risk estimation and model explanation.

Albumin and RDW, along with their static ratios, are well-established prognostic markers in critical illness. Low admission albumin predicts adverse outcomes after acute ischemic stroke/transient ischemic attack (12–15), and elevated RDW relates to 30-day mortality after intracerebral hemorrhage (16). Composite indices coupling inflammatory and nutritional signals (e.g., red cell distribution width-to–albumin ratio [RAR], C-reactive protein-to–albumin ratio [CAR]) improve discrimination across burn ICU, population cohorts, and elderly sepsis (6, 17–19); pairing RDW with nutritional scores (e.g., prognostic nutritional index [PNI]) also enhances stratification (20). A key limitation of these approaches, however, is that such measures are typically captured at a single time point. Even when short-horizon dynamics are considered, such as inflammatory/oxidative markers peaking around day 3 and tracking edema after ICH (21, 22), full longitudinal trajectories are rarely modeled. Our study addresses this gap by modeling longitudinal ARR trajectories (the inverse of RAR), which also provides a clinically intuitive directionality whereby higher ARR (higher albumin and/or lower RDW) aligns with a more favorable prognosis. Time-stratified Cox modeling accommodated proportional hazards (PH) violations, indicating that standardized ARR trajectories capture time-varying prognostic signals that single time-point scores may miss.

The ExtraTrees model showed favorable internal performance; calibration plots and decision-curve analyses suggested modest utility within selected risk ranges. Its discrimination was within the range reported for prior disease-specific approaches and commonly used static scores in ABI-related settings (23). Interpretability analyses (permutation- and loss-based importance and SHAP) consistently highlighted ventilation status, admission GCS, TBI, and ARR trajectory membership—features that are clinically plausible and commonly implicated in ABI prognostic modeling—while the inclusion of ARR trajectories adds longitudinal context beyond single time-point predictors (4, 24–26). Notably, external performance attenuation is consistent with domain shift across healthcare systems (e.g., differences in case mix, care processes, and laboratory measurement patterns), a limitation also reported in prior neurocritical ML studies and established scores with calibration drift across settings (27, 28). Therefore, use in new environments should be preceded by local validation and, when needed, recalibration or model updating.

Albumin is a negative acute-phase protein that sustains colloid oncotic pressure and provides antioxidant, anti-inflammatory, and ligand-transport functions. Low albumin integrates inflammation, malnutrition, and dysregulated volume status and has been linked to adverse outcomes after AIS/TIA and across cardiovascular conditions (12–14, 29). RDW reflects anisocytosis driven by oxidative stress, inflammation, impaired erythropoiesis, and inflammation-related iron dysregulation, including hypoferremia and iron-restricted red-cell production, thereby providing a plausible pathway linking critical illness to RDW elevation and prognostic information beyond anemia (30–32). RDW is associated with 30-day mortality after ICH (16), and its increase over time outperforms a single value for predicting death in elderly sepsis (21). Taken together, albumin (nutritional/oncotic) and RDW (erythropoietic/inflammatory) provide a biologic rationale for interpreting ARR as an integrated systemic signal. ARR is not brain-specific; it likely reflects the coupled systemic inflammatory–nutritional response accompanying ABI severity and its complications. Accordingly, the ARR phenotypes may reflect different balances between systemic stress and recovery over the ICU course: SHR reflects persistently unfavorable coupling (lower albumin and/or higher RDW), whereas SR reflects sustained recovery with improving albumin and relatively stable RDW. GD represents an intermediate, gradually deteriorating pattern, and RLD shows early improvement followed by a later downturn, compatible with a “second-hit” course. Importantly, these labels are descriptive and prognostic rather than prescriptive and should not be interpreted as directing specific neurocritical interventions.

After ABI, barrier disruption can trigger an early, tissue-injurious innate immune response with endothelial, pulmonary, and neuroinflammatory crosstalk, followed by later immune dysregulation and heightened infection susceptibility (33, 34). In ICH, inflammatory and oxidative markers peak around day 3 and track edema (22), supporting a multiphasic course in which systemic signals evolve over time. Against this background, our time-dependent associations are compatible with recovery-type ARR patterns marking a more favorable systemic trajectory, including attenuation of inflammatory and metabolic stress and improvement in nutritional or volume balance (35, 36). By contrast, the later excess risk observed in SHR may reflect sustained systemic stress and the accumulation of ICU complications. The exploratory early subgroup patterns by admission GCS and mechanical ventilation may reflect differences in baseline severity and acute-phase response, which can contribute to albumin suppression and RDW dysregulation. Thus, ARR trajectories may partly capture the systemic consequences of initial ABI severity rather than an isolated nutritional process. Nonetheless, as an observational study, residual confounding from unmeasured time-varying interventions and complication management cannot be excluded. Future prospective studies that capture time-varying treatments and complications in greater detail are needed to evaluate whether ARR trajectories can help identify patients at risk of potentially modifiable complications and whether trajectory-informed monitoring improves outcomes.

In practice, ARR trajectories may serve as longitudinal risk-stratification signals that support risk communication and monitoring prioritization, particularly in high-acuity subgroups defined by low admission GCS or mechanical ventilation. From a Clinical Nutrition perspective, unfavorable ARR trajectories may help identify patients who warrant closer nutrition-risk screening, repeated assessment of inflammatory and metabolic stress, and earlier dietetic review when interpreted together with established ICU nutrition assessment frameworks, neurological severity, organ dysfunction, feeding tolerance, infection status, fluid balance, and routine laboratory trends (37). These signals should be interpreted alongside standard ABI evaluation, including neurologic examinations, imaging, and physiologic trends, and should not be used as stand-alone triggers for albumin replacement, nutritional escalation, or other interventions. Given the attenuation in external validation, use in new settings requires local validation and may require threshold tuning, recalibration, or model updating to mitigate calibration drift and practice heterogeneity.

### Limitations

Several limitations should be acknowledged. First, the retrospective observational design leaves potential residual confounding from unmeasured time-varying treatments, ICU complications, nutritional support, fluid management, blood-product transfusion, albumin administration, hepatic function changes, and infection-related processes. Second, heterogeneity in laboratory timing, measurement frequency, ABI case mix, and care practices across cohorts may have affected ARR trajectory assignment, while missing-data assumptions may have further influenced trajectory modeling, particularly for smaller or imbalanced trajectory classes. Third, external validation showed attenuated performance, modest positive-class metrics, and a limited range of positive decision-curve benefit; therefore, the web-based research calculator should be regarded as an adjunctive risk-estimation tool pending prospective validation, local recalibration, and model updating, and ARR should be interpreted as a composite prognostic biomarker rather than a modifiable causal nutritional target.

## Conclusion

Standardized longitudinal ARR trajectories provide dynamic prognostic information in ABI and complement static assessments. An interpretable ExtraTrees model incorporating these trajectories showed favorable internal performance but attenuated external validation performance, supporting further evaluation as an adjunctive risk-estimation approach pending prospective validation and local recalibration.

## Data Availability

The datasets presented in this study can be found in online repositories. The names of the repository/repositories and accession number(s) can be found in the article/Supplementary material.
