# Supplementary material for: Dynamic albumin-to–RDW trajectories for time-dependent risk stratification in acute brain injury
Source: Front Nutr. 2026 Aug 17;13:1855636. doi: 10.3389/fnut.2026.1855636 (PMC13526662; doi:10.3389/fnut.2026.1855636)
Supplement: Supplementary file 1 [file Data_Sheet_1.PDF]

Supplementary Figure 1. Flowchart of Participant Selection and Study Workflow

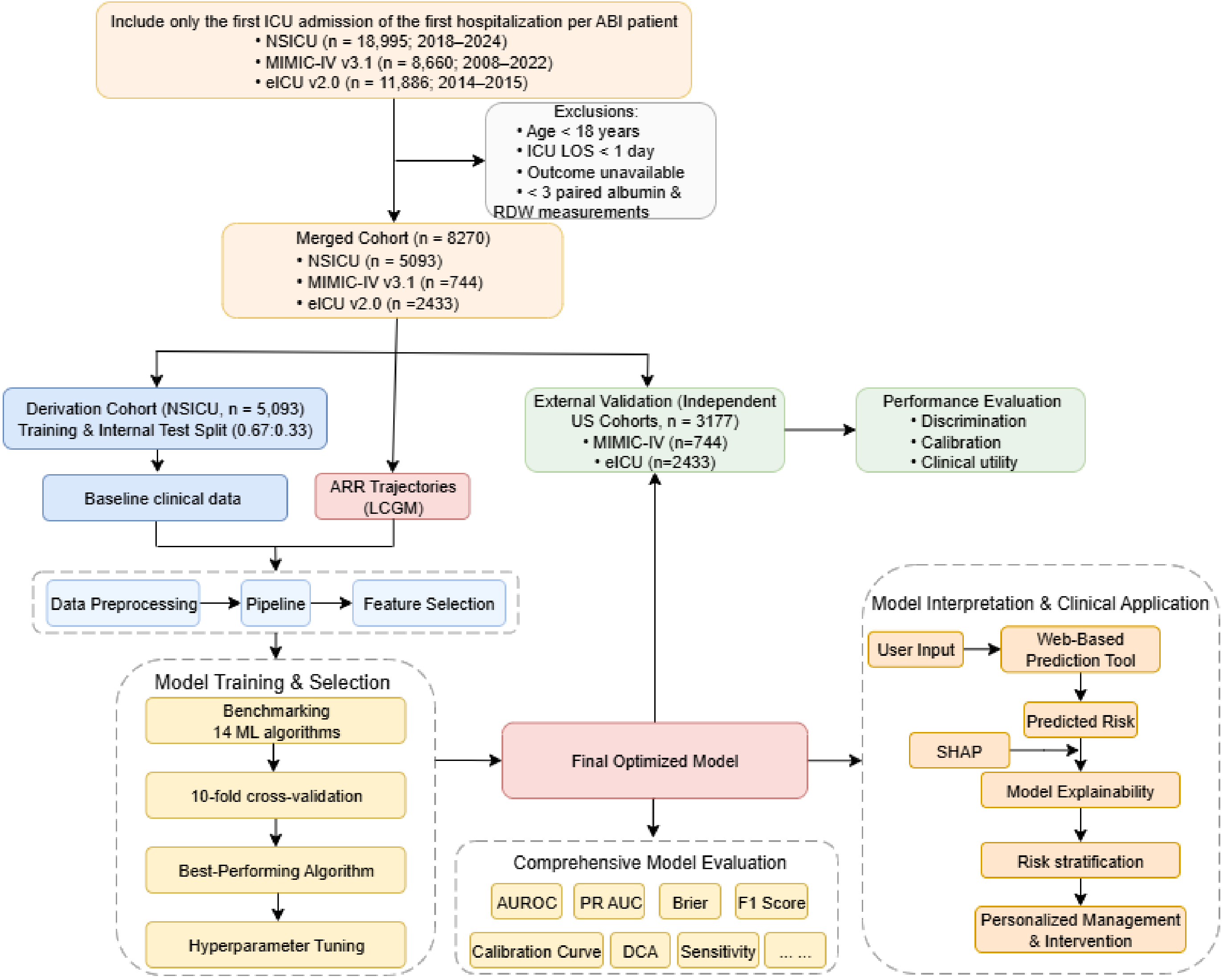

Legend: Multicenter data were harmonized and prespecified exclusions were applied. Albumin-to–red cell distribution width ratio (ARR) trajectories were estimated in the pooled cohort using latent class growth modeling (LCGM). The NSICU cohort was used for model development and internal validation, whereas MIMIC-IV and eICU served as independent external validation cohorts. Subsequent analyses included classifier benchmarking, final model selection and tuning, external validation, calibration and decision-curve assessment, SHAP-based interpretation, and deployment as a web-based research calculator for individualized risk estimation and model explanation.

Supplementary Table 1. Clinical Variables Extracted for Analysis

Category	Variables
**Demographic & admission**	Age (years); Sex (male/female); BMI (kg/m²); ICU type (neurological/non-neurological); TBI; Cerebral infarction; Admission type (elective/emergency); Admission time (day/night)
**Physiological parameters**	Temperature (°C); Respiratory rate (breaths/min); Heart rate (bpm); MAP (mmHg)
**Laboratory & derived measures**	Urine output (mL); RBC ($\times 10^{12}$/L); WBC ($\times 10^9$/L); Platelet ($\times 10^9$/L); ALT (U/L); BUN (mg/dL); Creatinine (mg/dL); Glucose (mg/dL); Sodium (mmol/L); Potassium (mmol/L); INR; **Albumin (g/L); RDW (%); Albumin/RDW ratio (ARR; original scale and z-score)**
**Comorbidities & severity**	Hypertension; Diabetes; Renal disease (CKD); Liver disease; CCI; SOFA; GCS at admission
**Treatments**	Ventilation; Vasopressor use; Mannitol use; Sedation; Dialysis; Craniotomy; Embolization; ICP monitoring
**Discharge disposition**	Discharge location (acute/ICU care; chronic/long-term care; home care/recovery)
**Outcomes & length of stay**	Hospital length of stay (days); ICU length of stay (days); In-hospital mortality

Notes: Baseline treatments within the first 24 hours after ICU admission included vasopressors, mannitol, and sedatives. Repeated measurements (albumin, RDW, and ARR on the original and z-score scales) within the trajectory window were summarized as mean, median, minimum, maximum, SD, and CV. Abbreviations: ICU, intensive care unit; MAP, mean arterial pressure; RBC, red blood cell count; WBC, white blood cell count; ALT, alanine aminotransferase; BUN, blood urea nitrogen; INR, international normalized ratio; CKD, chronic kidney disease; CCI, Charlson comorbidity index; SOFA, Sequential Organ Failure Assessment; GCS, Glasgow Coma Scale; ICP, intracranial pressure; RDW, red cell distribution width.

Supplementary Table 2. Missingness of Baseline Covariates in the Study Cohort

Variable	Missing, n	Missing, %
**Urine output, mL**	1717	20.76
**BMI, kg/m²**	1552	18.77
**ALT, U/L**	505	6.11

**GCS at admission**	390	4.72
**INR**	314	3.80
**WBC**	279	3.37
**BUN, mg/dL**	210	2.54
**Creatinine, mg/dL**	207	2.50
**Sodium, mmol/L**	196	2.37
**Temperature, °C**	133	1.61
**SOFA**	104	1.26
**Glucose, mg/dL**	99	1.20
**Respiratory rate, breaths/min**	39	0.47
**RBC, $\times 10^{12}$/L**	33	0.40
**MAP, mmHg**	31	0.37
**Platelet, $\times 10^{9}$/L**	28	0.34
**Heart rate, bpm**	24	0.29
**Potassium, mmol/L**	15	0.18
**Dialysis**	8	0.10
**Ventilation**	8	0.10

Notes: Missingness is reported as the number (n) and percentage (%) of patients with missing values for each covariate (before imputation). Covariates not shown had 0% missingness.

Supplementary Figure 2. Distributions of Observed and Imputed Values for Key Covariates

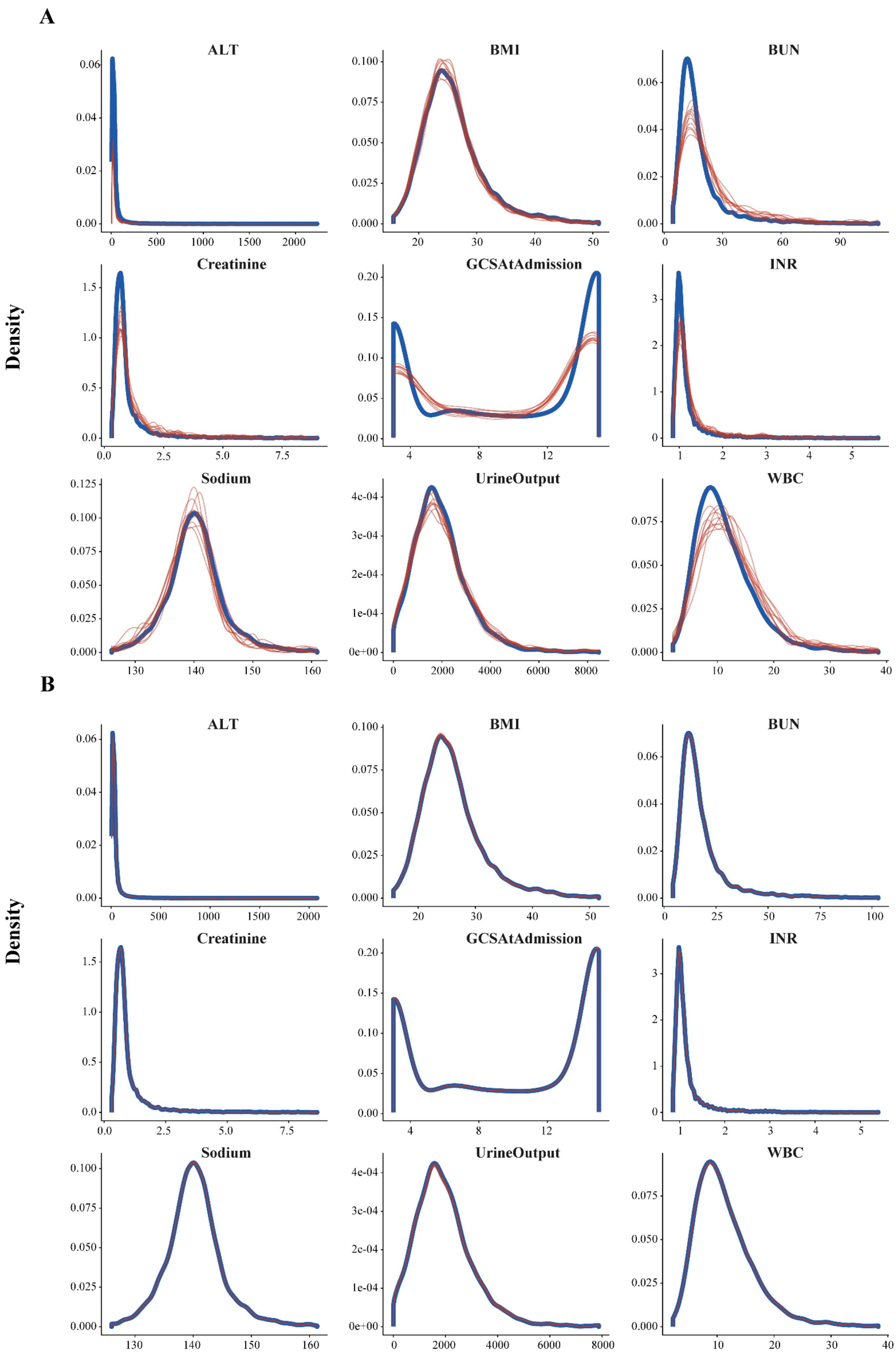
A
B
Density
ALT
BMI
BUN
Creatinine
GCSAtAdmission
INR
Sodium
UrineOutput
WBC

Legend: Panel A compares the distribution of observed values (thick blue line) with the distributions of imputed values for missing entries only (thin red lines; m = 10 imputed datasets). Panel B compares the observed distribution (thick blue line) with the distributions from each completed dataset (observed + imputed; thin red lines; m = 10). Distributions are shown as kernel density estimates. Only the nine covariates with the highest missingness are displayed for readability.

Supplementary Table 3. Model Fit Indices for ARR Trajectories

G	Log-Likelihood	AIC	BIC	SABIC	Entropy	Class Proportion (%)						Average Posterior Probability					
						1	2	3	4	5	6	1	2	3	4	5	6
2	-35925.69	71881.38	71986.69	71939.02	0.49	87.93	12.07					0.87	0.78				
3	-35830.88	71701.77	71842.17	71778.62	0.60	89.36	4.30	6.34				0.86	0.71	0.68			
4	-35800.62	71651.24	71826.75	71747.31	0.52	5.37	82.26	4.24	8.13			0.59	0.76	0.69	0.67		
5	-35765.29	71590.59	71801.20	71705.86	0.40	10.33	13.88	48.94	18.11	8.74		0.69	0.56	0.55	0.70	0.62	
6	-35743.40	71556.81	71802.52	71691.30	0.40	10.97	32.95	4.89	24.74	15.34	11.11	0.65	0.48	0.59	0.49	0.70	0.59

Note: LCGM was fitted to standardized ARR (z-score) trajectories in the merged ICU cohort (n=8,270). Model fit was evaluated using log-likelihood, AIC, BIC, and SABIC. Entropy and average posterior probabilities (APPs) summarize classification separation, and class proportions summarize the size of each latent class. Although information criteria improved with additional classes, the four-class solution (G=4) was selected for parsimony and interpretability.

Supplementary Table 4. Model Fit Indices for ARR Trajectories (Expanded Cohort; ≥2 Pairs)

G	Log-Likelihood	AIC	BIC	SABIC	Entropy	Class Proportion (%)						Average Posterior Probability					
						1	2	3	4	5	6	1	2	3	4	5	6
2	-38303.01	76636.03	76741.94	76694.28	0.74	94.02	5.98					0.95	0.79				
3	-38260.45	76560.91	76702.13	76638.57	0.38	64.80	11.88	23.33				0.72	0.56	0.70			
4	-38184.98	76419.95	76596.48	76517.03	0.51	8.35	79.26	4.60	7.79			0.60	0.76	0.70	0.66		
5	-38152.39	76364.79	76576.62	76481.28	0.41	9.10	14.11	48.37	19.40	9.02		0.71	0.59	0.56	0.70	0.62	
6	-38133.61	76337.21	76584.35	76473.12	0.41	9.90	32.53	6.42	22.97	16.60	11.58	0.67	0.48	0.60	0.49	0.69	0.58

Note: LCGM was fitted to standardized ARR (z-score) trajectories in the expanded cohort with ≥2 paired albumin–RDW measurements (n=8,613). Models with 2–6 classes were compared using log-likelihood, AIC, BIC, SABIC, entropy, class proportions, and APPs. The four-class solution (G=4) was selected based on overall fit, class size, and interpretability.

Supplementary Table 5. Model Fit Indices for ARR Trajectories Using 10 Sequential Measurements

G	Log-Likelihood	AIC	BIC	SABIC	Entropy	Class Proportion (%)						Average Posterior Probability					
						1	2	3	4	5	6	1	2	3	4	5	6
2	-38631.97	77293.93	77399.24	77351.57	0.57	90.41	9.59					0.89	0.78				
3	-38541.32	77122.63	77263.04	77199.48	0.65	91.08	2.79	6.13				0.88	0.68	0.66			
4	-38517.53	77085.06	77260.57	77181.13	0.60	2.83	2.35	87.91	6.92			0.67	0.61	0.82	0.65		
5	-38504.58	77069.17	77279.78	77184.44	0.41	5.70	18.69	53.47	11.78	10.36		0.68	0.70	0.59	0.57	0.60	
6	-38485.59	77041.18	77286.90	77175.68	0.41	5.21	12.97	41.79	17.99	5.60	16.43	0.67	0.70	0.53	0.49	0.55	0.55

Note: LCGM was fitted to standardized ARR (z-score) trajectories across up to 10 sequential paired measurements among patients with ≥3 paired albumin–RDW observations (n=8,270). Fit indices (log-likelihood, AIC, BIC, SABIC) and classification metrics (entropy, class proportions, APPs) are reported for 2–6 classes.

Supplementary Table 6. Model Fit Indices for Standardized Albumin Trajectories

G	Log-Likelihood	AIC	BIC	SABIC	Entropy	Class Proportion (%)						Average Posterior Probability					
						1	2	3	4	5	6	1	2	3	4	5	6
2	-38296.01	76622.02	76727.33	76679.66	0.68	90.62	9.38					0.93	0.79				
3	-38241.58	76523.16	76663.57	76600.01	0.36	55.03	16.41	28.56				0.69	0.64	0.69			
4	-38169.39	76388.79	76564.30	76484.85	0.43	16.31	67.75	7.55	8. 39			0.56	0.71	0.68	0.66		
5	-38147.00	76353.99	76564.60	76469.27	0.39	11.09	51.90	14.76	6.67	15.57		0.59	0.57	0.60	0.68	0.59	
6	-38113.93	76297.85	76543.57	76432.34	0.52	2.54	11.43	30.60	9.01	0.46	45.96	0.63	0.59	0.55	0.55	0.76	0.65

Note: LCGM was fitted to standardized albumin (z-score) trajectories in the merged ICU cohort (n=8,270). Model fit (log-likelihood, AIC, BIC, SABIC) and classification metrics (entropy, class proportions, APPs) are shown for 2–6 classes.

Supplementary Table 7. Model Fit Indices for Standardized RDW Trajectories

G	Log-Likelihood	AIC	BIC	SABIC	Entropy	Class Proportion (%)						Average Posterior Probability					
						1	2	3	4	5	6	1	2	3	4	5	6
2	-26462.80	52955.61	53060.91	53013.24	0.70	86.20	13.80					0.93	0.90				
3	-26106.23	52252.45	52392.86	52329.30	0.57	69.95	6.38	23.66				0.82	0.88	0.77			
4	-25911.82	51873.64	52049.15	51969.71	0.55	44.23	39.42	14.09	2.26			0.74	0.70	0.82	0.83		
5	-25812.93	51685.85	51896.47	51801.13	0.54	14.34	40.83	30.30	1.86	12.66		0.73	0.61	0.71	0.83	0.82	
6	-25759.44	51588.89	51834.60	51723.38	0.52	8.62	29.88	29.73	20.73	1.46	9.58	0.74	0.58	0.58	0.69	0.82	0.80

Note: LCGM was fitted to standardized RDW (z-score) trajectories in the merged ICU cohort (n=8,270). Model fit (log-likelihood, AIC, BIC, SABIC) and classification metrics (entropy, class proportions, APPs) are shown for 2–6 classes.

Supplementary Figure 3. Four ARR Trajectory Phenotypes in the Expanded Cohort

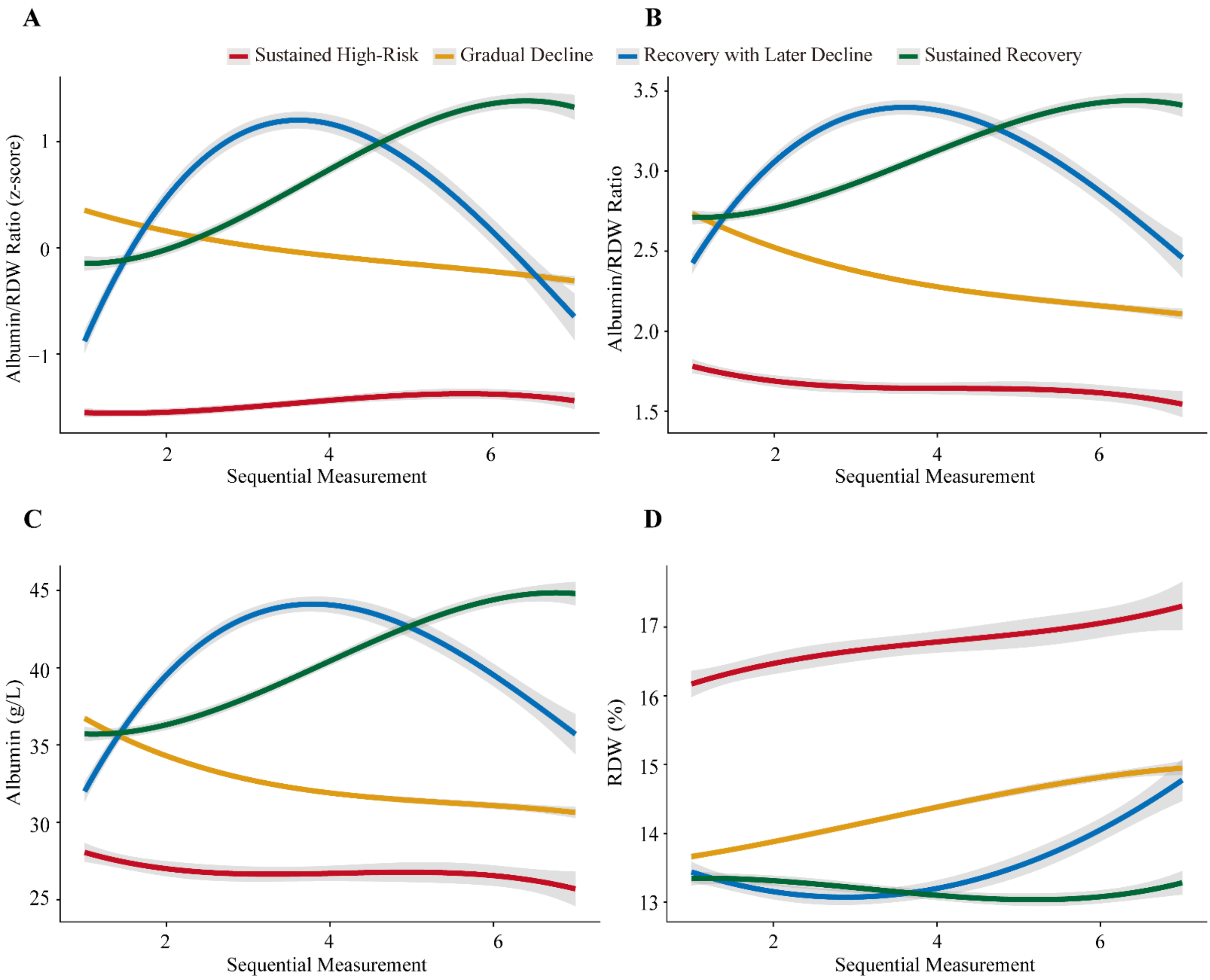

Legend: Panels A–D show trajectories across sequential measurements for (A) standardized ARR (z-score), (B) ARR, (C) albumin, and (D) RDW in the expanded cohort (n=8,613; ≥2 paired albumin–RDW measurements). Solid lines indicate model-estimated mean trajectories and shaded bands represent 95% CIs. Colors denote trajectory phenotypes: Sustained High-Risk (red), Gradual Decline (orange), Recovery with Later Decline (blue), and Sustained Recovery (green).

Supplementary Figure 4. Four ARR Trajectory Phenotypes Using 10 Sequential Measurements

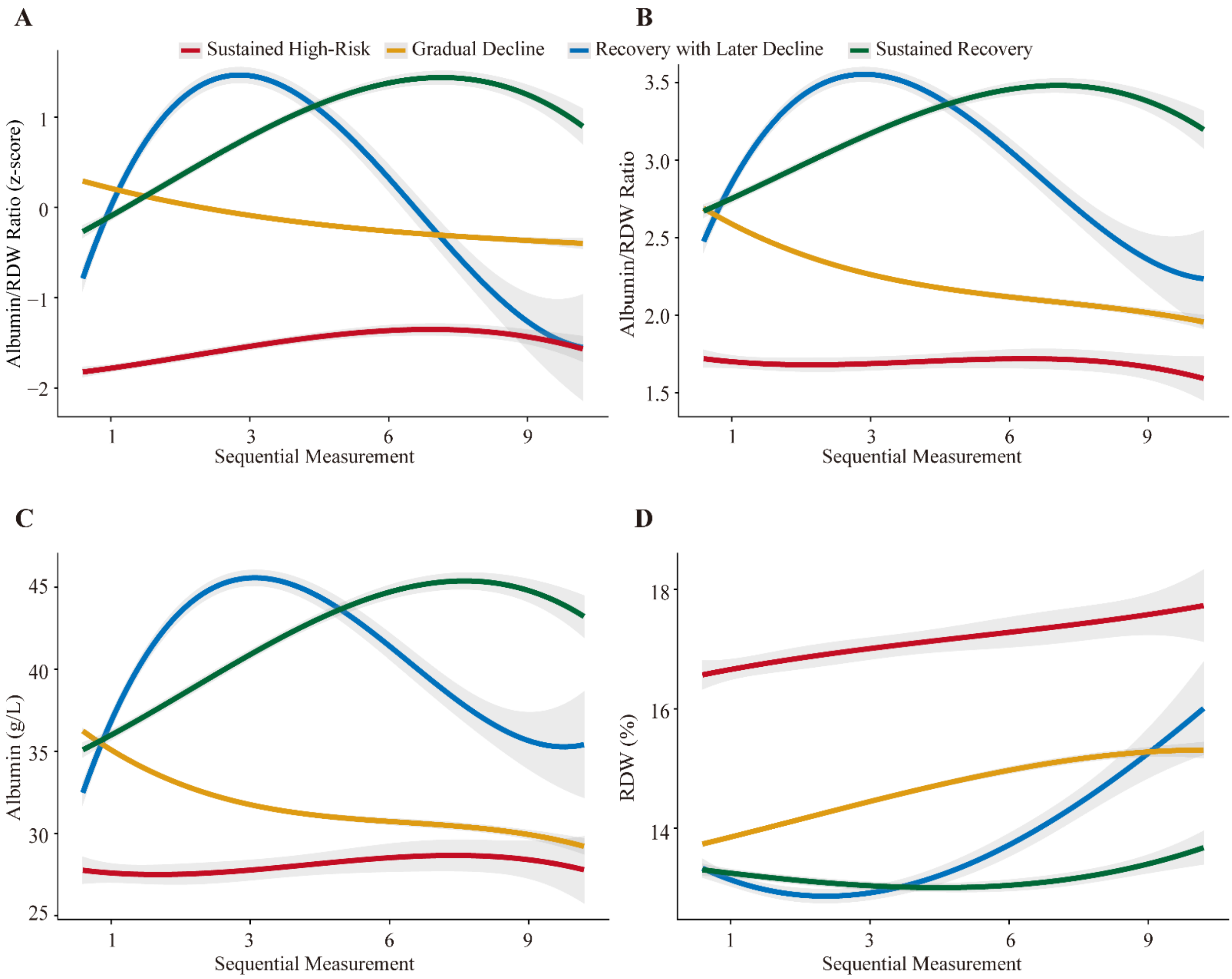

Legend: Panels A–D show trajectories across 10 sequential measurements for (A) standardized ARR (z-score), (B) ARR, (C) albumin, and (D) RDW in the primary cohort (n=8,270; ≥3 paired albumin–RDW measurements). Solid lines indicate model-estimated mean trajectories and shaded bands represent 95% CIs. Colors denote trajectory phenotypes: Sustained High-Risk (red), Gradual Decline (orange), Recovery with Later Decline (blue), and Sustained Recovery (green).

Supplementary Figure 5. Four-Class Trajectories of Albumin and RDW

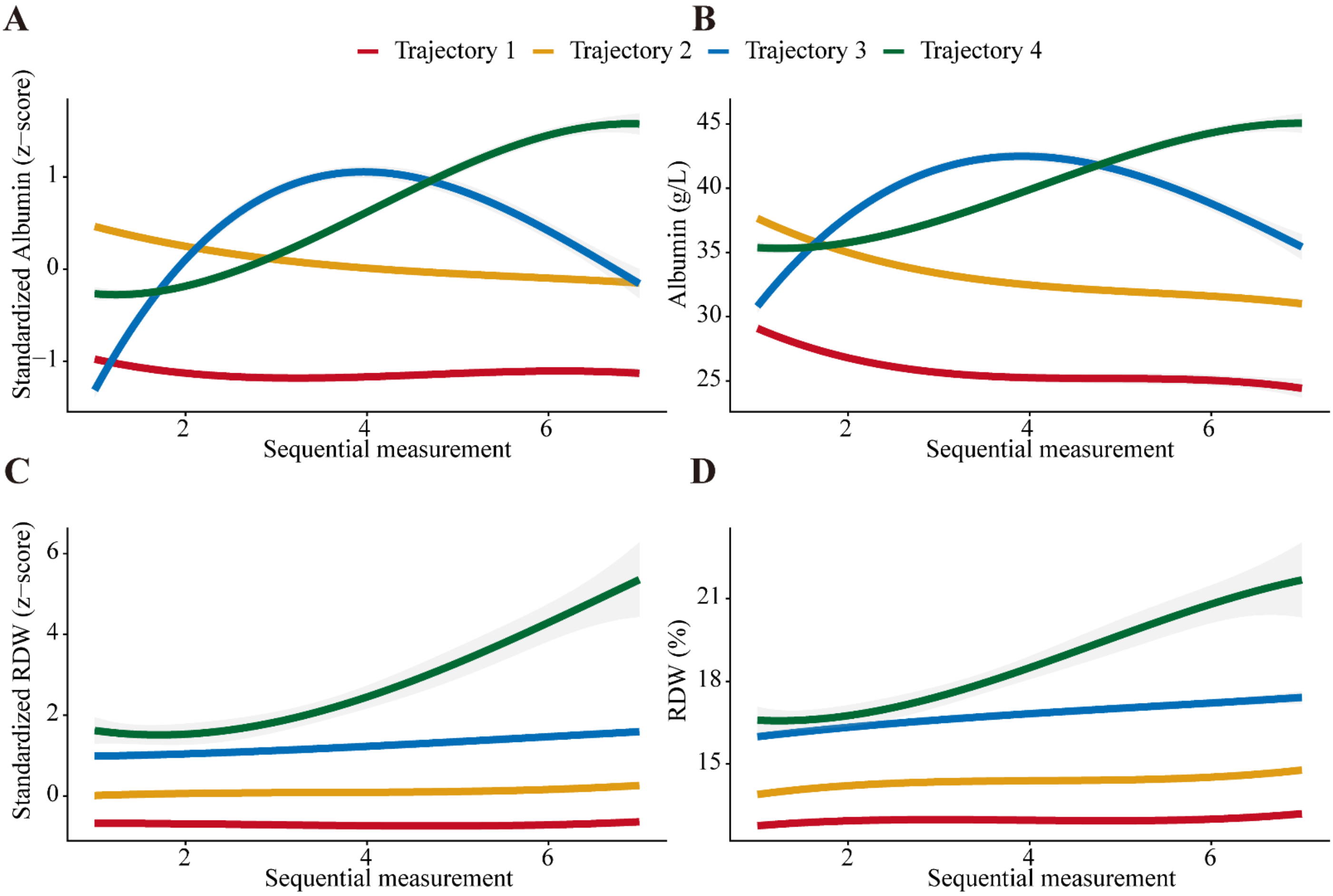

Legend: Panels A–B show the four latent classes from an LCGM fitted to standardized albumin trajectories (A, z-score; B, g/L). Panels C–D show the four latent classes from a separate LCGM fitted to standardized RDW trajectories (C, z-score; D, %). Solid lines indicate class-specific fitted means and shaded bands denote 95% confidence intervals. Trajectory labels (1–4) are model-specific and do not imply a one-to-one correspondence between the albumin and RDW classes.

Supplementary Figure 6. Quantitative Profiles and Discharge Disposition by ARR Trajectories

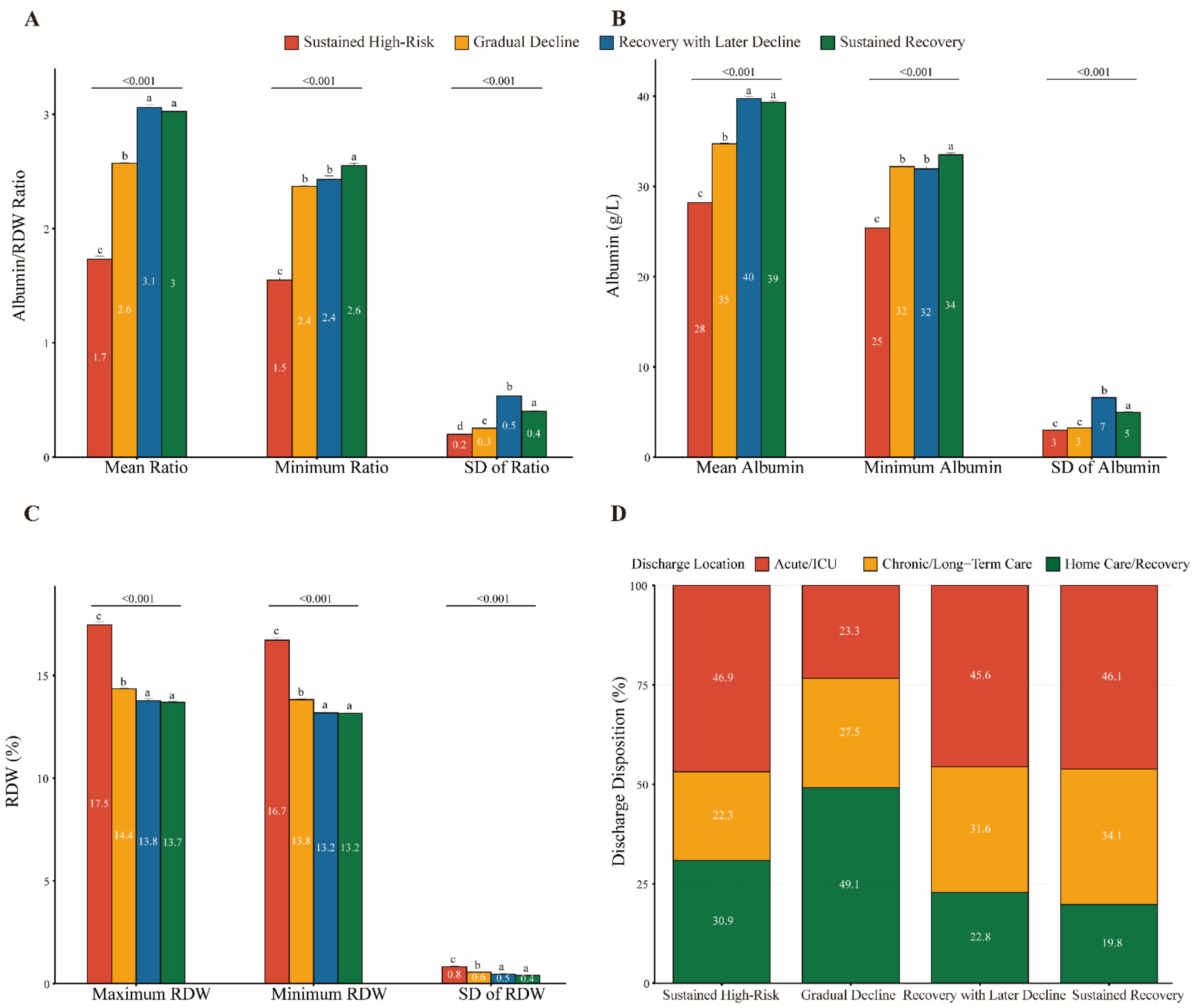

Legend: Panels A–C summarize ARR, albumin, and red cell distribution width metrics across the four ARR trajectory phenotypes (group means). Omnibus P values are shown above brackets; different lowercase letters indicate multiplicity-adjusted pairwise differences (P<0.05). Panel D shows discharge disposition by phenotype; stacked-bar colors denote disposition category (acute/ICU care, chronic/long-term care, home care/recovery).

Supplementary Table 8. Additional Baseline Characteristics and Management by ARR Trajectory

Characteristics	Total (n = 8270)	Sustained High-Risk (n = 444)	Gradual Decline (n = 6803)	Recovery with Later Decline (n = 351)	Sustained Recovery (n = 672)	*P*
**Sex, male**	4765 (57.6)	232 (52.3)	3914 (57.5)	196 (55.8)	423 (62.9)	0.004
**BMI, kg/m²**	25.1 (22.4, 28.4)	24.6 (21.5, 28.2)	25.2 (22.5, 28.7)	23.5 (21.2, 26.0)	24.6 (22.0, 27.4)	<0.001
**Admission type**	5160 (62.4)	269 (60.6)	4121 (60.6)	274 (78.1)	496 (73.8)	<0.001
**Admission time**						<0.001
**Day shift**	5068 (61.3)	260 (58.6)	4303 (63.3)	159 (45.3)	346 (51.5)	
**Night shift**	3202 (38.7)	184 (41.4)	2500 (36.7)	192 (54.7)	326 (48.5)	
**Cerebral infarction**	1610 (19.5)	86 (19.4)	1416 (20.8)	36 (10.3)	72 (10.7)	<0.001
**Respiratory rate, breaths/min**	20.0 ± 9.0	20.6 ± 9.1	20.2 ± 9.3	18.0 ± 5.7	18.7 ± 7.0	<0.001
**MAP, mmHg**	96.9 ± 27.7	90.7 ± 28.4	97.2 ± 28.4	96.6 ± 20.7	98.1 ± 22.2	<0.001
**Urine output, mL**	1980.3 ± 1276.8	1976.5 ± 1234.9	1983.5 ± 1301.4	1963.8 ± 1076.6	1962.7 ± 1176.8	0.977
**WBC, ×10⁹/L**	11.5 ± 6.3	12.1 ± 7.8	11.4 ± 6.4	11.6 ± 5.3	12.1 ± 5.2	0.016
**ALT, U/L**	20.5 (13.9, 34.4)	20.4 (13.0, 35.0)	21.0 (14.0, 35.4)	17.4 (13.0, 28.4)	18.0 (12.8, 29.0)	<0.001
**Creatinine, mg/dL**	0.7 (0.6, 1.0)	0.8 (0.6, 1.4)	0.7 (0.6, 1.0)	0.7 (0.6, 0.9)	0.7 (0.6, 0.9)	<0.001
**Potassium, mmol/L**	3.8 ± 0.5	3.9 ± 0.6	3.8 ± 0.5	3.8 ± 0.5	3.8 ± 0.5	<0.001
**INR**	1.2 ± 0.7	1.4 ± 0.8	1.2 ± 0.7	1.2 ± 0.5	1.1 ± 0.6	<0.001
**Hypertension**	4814 (58.2)	261 (58.8)	3922 (57.7)	187 (53.3)	444 (66.1)	<0.001
**Diabetes**	1422 (17.2)	96 (21.6)	1150 (16.9)	54 (15.4)	122 (18.2)	0.051
**Liver disease**	1363 (16.5)	93 (20.9)	970 (14.3)	114 (32.5)	186 (27.7)	<0.001
**Dialysis**	234 (2.8)	36 (8.1)	185 (2.7)	7 (2)	6 (0.9)	<0.001
**ICP monitoring**	1455 (17.6)	81 (18.2)	1102 (16.2)	109 (31.1)	163 (24.3)	<0.001
**Sedation**	2736 (33.1)	161 (36.3)	2028 (29.8)	199 (56.7)	348 (51.8)	<0.001
**Mannitol**	1765 (21.3)	105 (23.6)	1230 (18.1)	157 (44.7)	273 (40.6)	<0.001

**GCS at discharge**	11.4 ± 4.2	9.7 ± 4.7	11.8 ± 4.1	9.8 ± 3.9	9.7 ± 3.8	<0.001
**Discharge location**						<0.001
**Acute/ICU care**	2266 (27.4)	208 (46.8)	1588 (23.3)	160 (45.6)	310 (46.1)	
**Chronic/Long-Term Care**	2310 (27.9)	99 (22.3)	1871 (27.5)	111 (31.6)	229 (34.1)	
**Home Care/Recovery**	3694 (44.7)	137 (30.9)	3344 (49.2)	80 (22.8)	133 (19.8)	
**Hospital length of stay, days**	12.3 (8.0, 18.8)	14.0 (8.0, 21.0)	12.0 (7.7, 18.0)	15.0 (12.0, 20.0)	16.0 (13.0, 21.0)	<0.001
**In-hospital mortality**	1209 (14.6)	129 (29.1)	971 (14.3)	37 (10.5)	72 (10.7)	<0.001
**ICU length of stay, days**	7.0 (2.9, 14.2)	8.6 (3.5, 16.0)	5.8 (2.7, 12.5)	13.4 (9.7, 18.2)	14.7 (11.1, 19.3)	<0.001

Notes: Continuous variables are summarized as mean ± SD or median (IQR), as appropriate; categorical variables as n (%). Trajectory classes: 1 (Sustained High-Risk), 2 (Gradual Decline), 3 (Recovery with Later Decline), and 4 (Sustained Recovery). **Abbreviations:** BMI, body mass index; MAP, mean arterial pressure; WBC, white blood cell count; ALT, alanine aminotransferase; INR, international normalized ratio; ICP, intracranial pressure.

Supplementary Table 9. Baseline Characteristics of Patients With Two Versus ≥3 Paired Albumin–RDW Measurements

Characteristics	Total (≥2 pairs) (n=8,613)	Excluded (2 pairs) (n=343)	Included (≥3 pairs) (n=8,270)	*P*
**Age, years**	59.0 ± 15.4	62.4 ± 17.0	58.9 ± 15.4	<0.001
**TBI**	2223 (25.8)	87 (25.4)	2136 (25.8)	0.847
**Temperature, °C**	36.6 ± 0.8	36.6 ± 0.6	36.6 ± 0.8	0.910
**Heart rate, bpm**	88.0 ± 24.3	88.9 ± 25.7	88.0 ± 24.3	0.518
**RBC, $\times 10^{12}$/L**	3.8 ± 0.8	3.8 ± 0.8	3.8 ± 0.8	0.937
**Platelet, $\times 10^{9}$/L**	185.2 ± 79.5	189.5 ± 82.4	185.0 ± 79.4	0.309
**BUN, mg/dL**	14.6 (10.9, 20.4)	15.4 (11.2, 23.0)	14.5 (10.9, 20.2)	0.040
**Glucose, mg/dL**	138.0 ± 67.0	134.4 ± 61.7	138.1 ± 67.2	0.328
**Sodium, mmol/L**	140.3 ± 5.4	139.4 ± 4.6	140.4 ± 5.4	0.001
**CKD**	1253 (14.5)	35 (10.2)	1218 (14.7)	0.02
**CCI**	3.0 (2.0, 5.0)	3.0 (2.0, 5.0)	3.0 (2.0, 5.0)	0.832
**SOFA**	4.0 (2.0, 6.0)	4.0 (2.0, 6.0)	4.0 (2.0, 6.0)	0.095
**GCS at admission**	10.0 ± 5.1	12.9 ± 3.4	9.9 ± 5.1	<0.001
**Ventilation**	3572 (41.5)	60 (17.6)	3512 (42.5)	<0.001
**Embolization**	2832 (32.9)	77 (22.4)	2755 (33.3)	<0.001
**Craniotomy**	2645 (30.7)	67 (19.5)	2578 (31.2)	<0.001
**Vasopressor use**	1285 (14.9)	35 (10.2)	1250 (15.1)	0.012
**Sex, male**	4943 (57.4)	178 (51.9)	4765 (57.6)	0.036
**BMI, kg/m²**	25.1 (22.4, 28.4)	25.4 (22.0, 30.2)	25.1 (22.4, 28.4)	0.266
**Admission type**	5315 (61.7)	155 (45.2)	5160 (62.4)	<0.001
**Admission time**				<0.001
**Day shift**	5315 (61.7)	247 (72)	5068 (61.3)	
**Night shift**	3298 (38.3)	96 (28)	3202 (38.7)	
**Cerebral infarction**	1733 (20.1)	123 (35.9)	1610 (19.5)	<0.001
**Respiratory rate, breaths/min**	20.1 ± 9.1	21.4 ± 10.7	20.0 ± 9.0	0.005
**MAP, mmHg**	96.8 ± 27.9	94.3 ± 32.0	96.9 ± 27.7	0.098
**Urine output, mL**	1978.4 ± 1271.5	1926.1 ± 1110.8	1980.3 ± 1276.8	0.524
**WBC, $\times 10^{9}$/L**	11.5 ± 7.5	12.1 ± 22.0	11.5 ± 6.3	0.183
**ALT, U/L**	20.7 (13.9, 34.5)	23.0 (15.2, 36.0)	20.5 (13.9, 34.4)	0.055
**Creatinine, mg/dL**	0.7 (0.6, 1.0)	0.8 (0.6, 1.1)	0.7 (0.6, 1.0)	0.004
**Potassium, mmol/L**	3.8 ± 0.5	3.7 ± 0.5	3.8 ± 0.5	0.006
**INR**	1.2 ± 0.7	1.3 ± 0.7	1.2 ± 0.7	0.123
**Hypertension**	4996 (58.0)	182 (53.1)	4814 (58.2)	0.058
**Diabetes**	1481 (17.2)	59 (17.2)	1422 (17.2)	0.998
**Liver disease**	1380 (16.0)	17 (5)	1363 (16.5)	<0.001
**Dialysis**	241 (2.8)	7 (2.1)	234 (2.8)	0.393
**ICP monitoring**	1465 (17.0)	10 (2.9)	1455 (17.6)	<0.001
**Sedation**	2792 (32.4)	56 (16.3)	2736 (33.1)	<0.001
**Mannitol**	1781 (20.7)	16 (4.7)	1765 (21.3)	<0.001
**GCS at discharge**	11.5 ± 4.2	13.8 ± 2.4	11.4 ± 4.2	<0.001
**Discharge location**				<0.001

**Acute/ICU care**	2284 (26.5)	18 (5.2)	2266 (27.4)	
**Chronic/Long-Term Care**	2418 (28.1)	108 (31.5)	2310 (27.9)	
**Home Care/Recovery**	3911 (45.4)	217 (63.3)	3694 (44.7)	
**Hospital length of stay, days**	12.1 (8.0, 18.4)	10.4 (7.5, 16.2)	12.3 (8.0, 18.8)	0.002
**In-hospital mortality**	1228 (14.3)	19 (5.5)	1209 (14.6)	<0.001
**ICU length of stay, days**	6.7 (2.8, 13.8)	2.4 (1.6, 3.9)	7.0 (2.9, 14.2)	<0.001

Notes: Data are mean ± SD, median (IQR), or n (%). P values compare Excluded (2 pairs) vs Included (≥3 pairs). Abbreviations: ALT, alanine aminotransferase; BMI, body mass index; BUN, blood urea nitrogen; CCI, Charlson comorbidity index; CKD, chronic kidney disease; GCS, Glasgow Coma Scale; ICP, intracranial pressure; ICU, intensive care unit; INR, international normalized ratio; MAP, mean arterial pressure; RBC, red blood cell count; RDW, red cell distribution width; SOFA, Sequential Organ Failure Assessment; TBI, traumatic brain injury; WBC, white blood cell count.

Supplementary Figure 7. Proportional Hazards Diagnostics for Cox Models

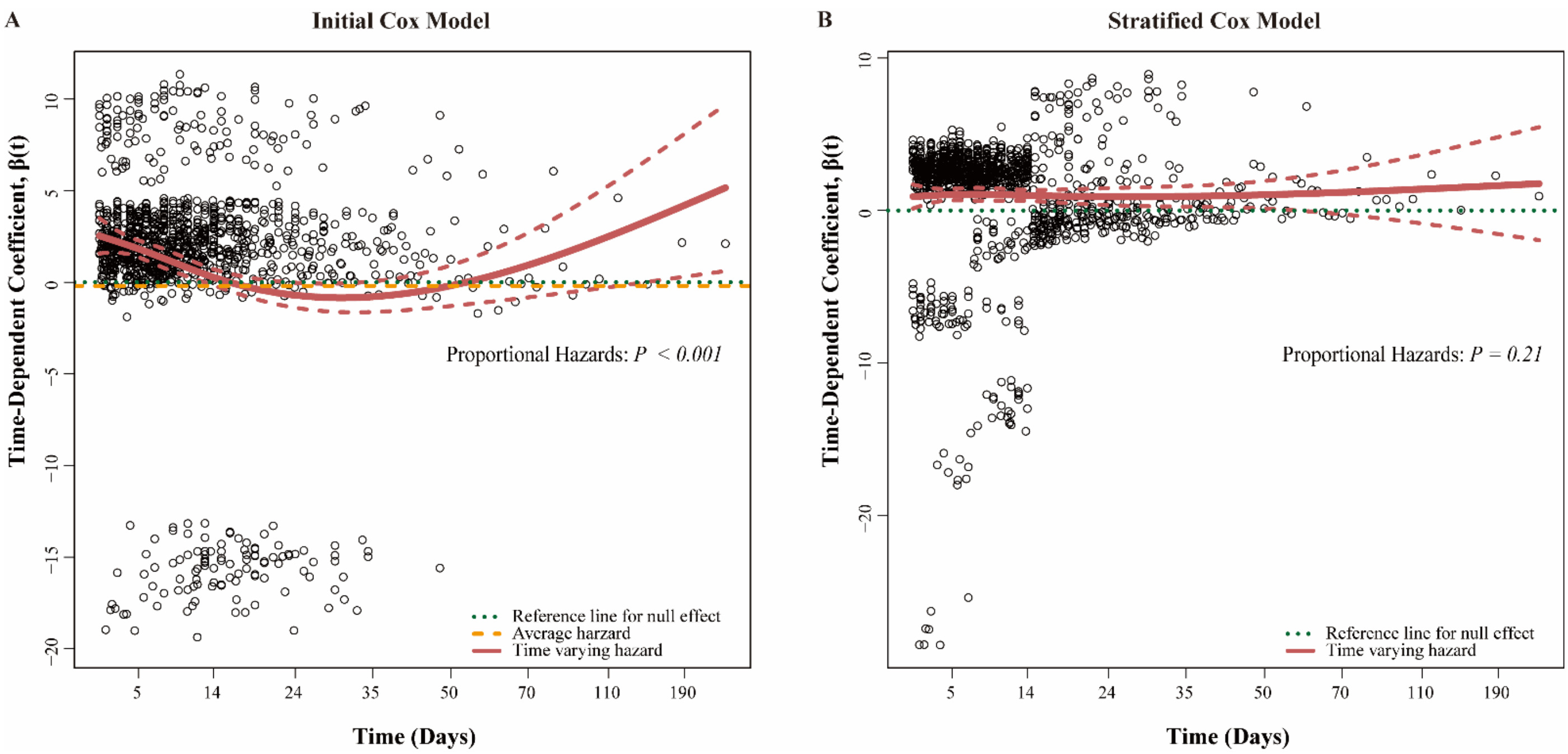

Legend: Scaled Schoenfeld residuals for indicators of ARR trajectory class membership. Smoothed time-varying coefficient functions β(t) are shown with 95% confidence bands. (A) The unstratified Cox model shows evidence of non-PH (global test P <0.001). (B) The time-stratified Cox model (0–7, 7–14, and >14 days) shows improved PH behavior (global test P = 0.21).

Supplementary Table 10. Sensitivity Analysis Using Alternative Time Cutpoints (5-Day Intervals)

Time interval	Model 1		Model 2		Model 3	
	HR (95% CI)	*P*	HR (95% CI)	*P*	HR (95% CI)	*P*
**0–5 d**						
**Gradual Decline**	Ref		Ref		Ref	
**Sustained High-Risk**	2.17 (1.58–2.97)	<0.001	1.66 (1.20–2.29)	0.002	1.39 (1.01–1.91)	0.047
**Recovery with Later Decline**	0.18 (0.06–0.55)	0.003	0.20 (0.06–0.61)	0.005	0.15 (0.05–0.47)	0.001
**Sustained Recovery**	0.19 (0.08–0.42)	<0.001	0.21 (0.09–0.47)	<0.001	0.17 (0.07–0.37)	<0.001
**5–10 d**						
**Gradual Decline**	Ref		Ref		Ref	
**Sustained High-Risk**	1.45 (0.97–2.17)	0.070	1.10 (0.73–1.65)	0.648	0.94 (0.62–1.41)	0.749
**Recovery with Later Decline**	0.41 (0.19–0.87)	0.019	0.46 (0.22–0.99)	0.046	0.37 (0.17–0.78)	0.009
**Sustained Recovery**	0.21 (0.10–0.44)	<0.001	0.24 (0.11–0.50)	<0.001	0.18 (0.09–0.39)	<0.001
**10–15 d**						
**Gradual Decline**	Ref		Ref		Ref	
**Sustained High-Risk**	1.64 (1.03–2.62)	0.036	1.34 (0.84–2.14)	0.226	1.12 (0.70–1.79)	0.635
**Recovery with Later Decline**	0.73 (0.37–1.43)	0.356	0.85 (0.43–1.67)	0.634	0.70 (0.35–1.37)	0.294
**Sustained Recovery**	0.89 (0.57–1.38)	0.596	1.06 (0.69–1.65)	0.785	0.86 (0.56–1.34)	0.513
**>15 d**						
**Gradual Decline**	Ref		Ref		Ref	
**Sustained High-Risk**	1.63 (1.16–2.30)	0.005	1.40 (0.99–1.97)	0.057	1.42 (1.01–2.01)	0.043
**Recovery with Later Decline**	1.22 (0.76–1.98)	0.410	1.65 (1.02–2.69)	0.043	1.47 (0.90–2.39)	0.120
**Sustained Recovery**	1.15 (0.81–1.64)	0.438	1.45 (1.02–2.07)	0.040	1.23 (0.86–1.75)	0.259

Note: Stratified Cox models were fit using alternative 5-day cutpoints (0–5, 5–10, 10–15, and >15 days). ARR trajectory phenotype was the exposure with Gradual Decline as the reference. Entries are HRs (95% CIs) with two-sided P values. Model 1 unadjusted; Model 2 adjusted for age, ICU type, TBI, temperature, heart rate, RBC, platelet, BUN, glucose, sodium, CKD, and CCI; Model 3 further adjusted for SOFA, GCS at admission, ventilation, embolization, craniotomy, and vasopressor use.

Supplementary Table 11. RMST Comparisons Across ARR Trajectories at Prespecified Time Horizons

Time interval	Difference in RMST, days						RMST ratio					
	Model 1		Model 2		Model 3		Model 1		Model 2		Model 3	
	Estimate (95% CI)	*P*	Estimate (95% CI)	*P*	Estimate (95% CI)	*P*	Estimate (95% CI)	*P*	Estimate (95% CI)	*P*	Estimate (95% CI)	*P*
**7 days**												
**Gradual Decline**	Ref		Ref		Ref		Ref		Ref		Ref	
**Sustained High-Risk**	-0.22 (-0.35–-0.10)	<0.001	-0.15 (-0.26–-0.03)	0.014	-0.08 (-0.19–0.04)	0.191	0.97 (0.95–0.99)	<0.001	0.98 (0.96–1.00)	0.016	0.99 (0.97–1.01)	0.196
**Recovery with Later Decline**	0.16 (0.12–0.20)	<0.001	0.13 (0.08–0.18)	<0.001	0.19 (0.13–0.25)	<0.001	1.02 (1.02–1.03)	<0.001	1.02 (1.01–1.03)	<0.001	1.03 (1.02–1.04)	<0.001
**Sustained Recovery**	0.16 (0.12–0.20)	<0.001	0.14 (0.10–0.18)	<0.001	0.19 (0.14–0.24)	<0.001	1.02 (1.02–1.03)	<0.001	1.02 (1.01–1.03)	<0.001	1.03 (1.02–1.04)	<0.001
**14 days**												
**Gradual Decline**	Ref		Ref		Ref		Ref		Ref		Ref	
**Sustained High-Risk**	-0.71 (-1.05–-0.37)	<0.001	-0.57 (-0.91–-0.23)	<0.001	-0.38 (-0.72–-0.05)	0.025	0.95 (0.92–0.97)	<0.001	0.96 (0.93–0.98)	0.001	0.97 (0.94–1.00)	0.027
**Recovery with Later Decline**	0.61 (0.45–0.78)	<0.001	0.43 (0.24–0.62)	<0.001	0.58 (0.37–0.79)	<0.001	1.05 (1.03–1.06)	<0.001	1.03 (1.02–1.05)	<0.001	1.04 (1.03–1.06)	<0.001
**Sustained Recovery**	0.68 (0.56–0.80)	<0.001	0.50 (0.37–0.63)	<0.001	0.63 (0.48–0.77)	<0.001	1.05 (1.04–1.06)	<0.001	1.04 (1.03–1.05)	<0.001	1.05 (1.04–1.06)	<0.001
**28 days**												
**Gradual Decline**	Ref		Ref		Ref		Ref		Ref		Ref	
**Sustained High-Risk**	-2.39 (-3.33–-1.44)	<0.001	-2.18 (-3.19–-1.16)	<0.001	-1.77 (-2.81–-0.74)	<0.001	0.90 (0.86–0.94)	<0.001	0.91 (0.87–0.95)	<0.001	0.93 (0.88–0.97)	<0.001
**Recovery with Later Decline**	1.24 (0.51–1.97)	<0.001	0.61 (-0.21–1.43)	0.147	0.77 (-0.14–1.67)	0.096	1.05 (1.02–1.08)	<0.001	1.02 (0.99–1.06)	0.151	1.03 (0.99–1.07)	0.096
**Sustained Recovery**	1.67 (1.18–2.16)	<0.001	1.46 (0.88–2.03)	<0.001	1.68 (1.01–2.36)	<0.001	1.07 (1.05–1.09)	<0.001	1.06 (1.04–1.08)	<0.001	1.07 (1.04–1.10)	<0.001

Notes: Estimates are RMST differences (trajectory class − Gradual Decline) and RMST ratios (trajectory class/Gradual Decline) at $\tau = 7$, 14, and 28 days, reported as estimate (95%

CI) with two-sided P values. Gradual Decline was used as the reference trajectory. Model 1 was unadjusted. Model 2 adjusted for age, ICU type, TBI, temperature, heart rate, RBC, platelets, BUN, glucose, sodium, CKD, and CCI. Model 3 additionally adjusted for SOFA, GCS at admission, mechanical ventilation, embolization, craniotomy, and vasopressor

Supplementary Figure 8. RMST Across Prespecified Horizons by ARR Trajectory

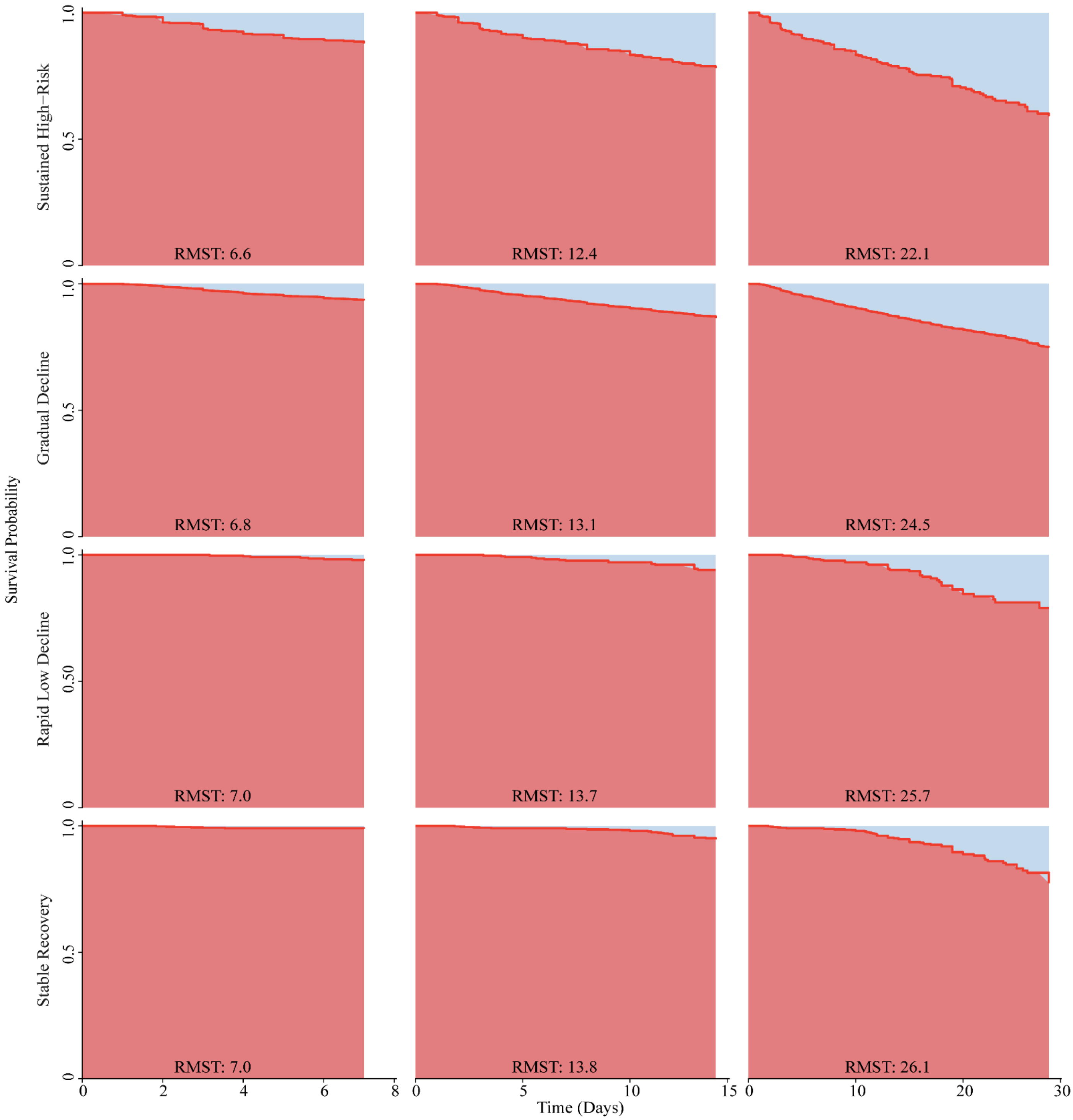

Legend: Kaplan–Meier survival curves and restricted mean survival time (RMST) estimates are shown for each ARR trajectory at τ = 7, 14, and 28 days. Shaded areas denote RMST up to the prespecified time horizon.

Supplementary Figure 9. High-Confidence Trajectory Assignment Sensitivity Analysis

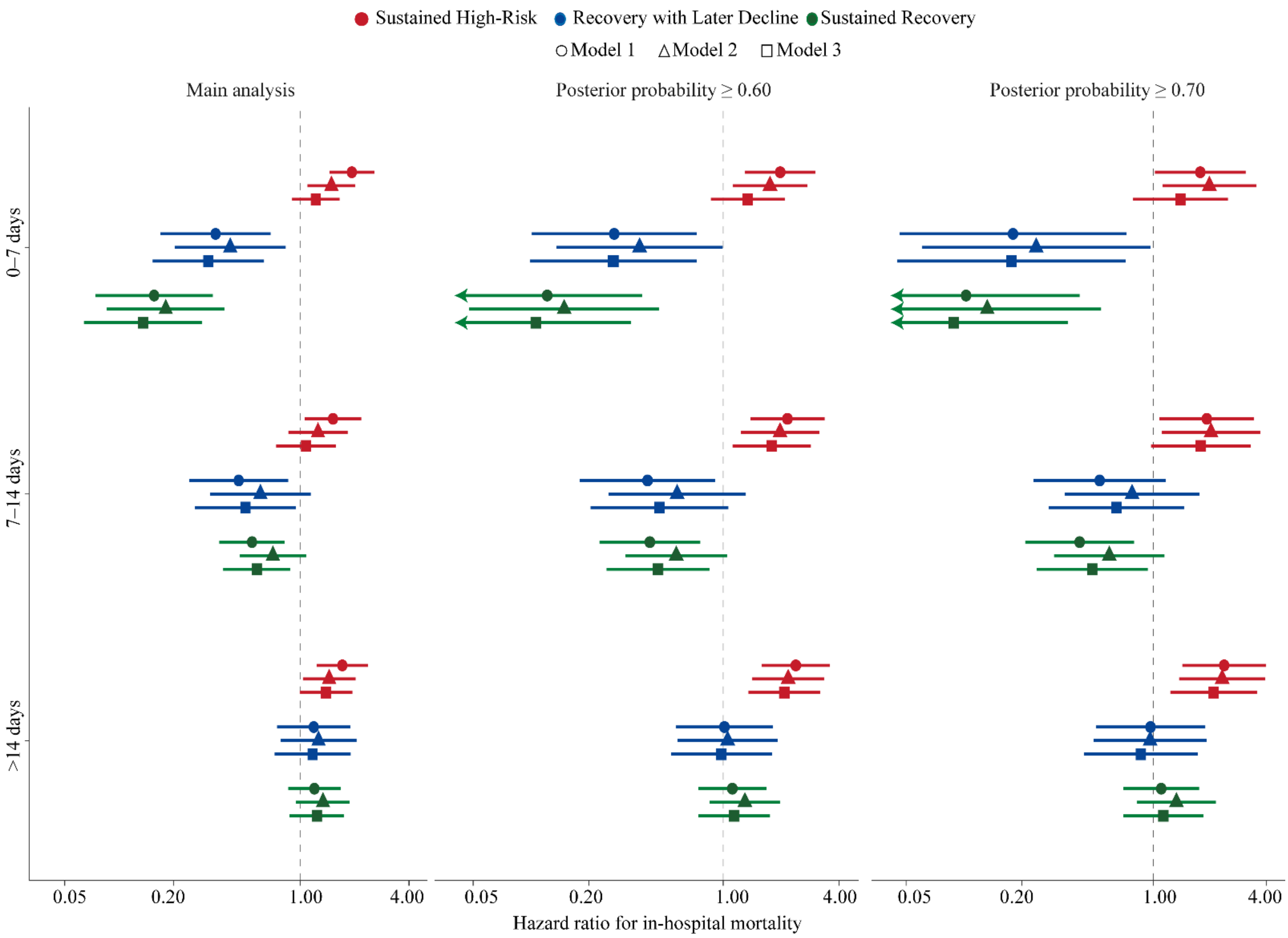

Legend: Forest plots show time-stratified hazard ratios for in-hospital mortality in the main cohort and high-confidence posterior probability subsets. High-confidence subsets were defined as assigned posterior probability ≥ 0.60 or ≥ 0.70. Gradual Decline was the reference trajectory. Colors indicate ARR trajectory phenotypes, and point shapes indicate adjustment models. Model 1 was unadjusted; Model 2 adjusted for age, ICU type, TBI, temperature, heart rate, RBC, platelets, BUN, glucose, sodium, CKD, and CCI; Model 3 further adjusted for SOFA, GCS at admission, mechanical ventilation, embolization, craniotomy, and vasopressor use. Arrows indicate that the lower limit of the 95% CI extends beyond the plotted lower axis limit of 0.05.

Supplementary Figure 10. Hazard Ratios by ARR Trajectory Stratified by Time Interval in Additional Subgroups

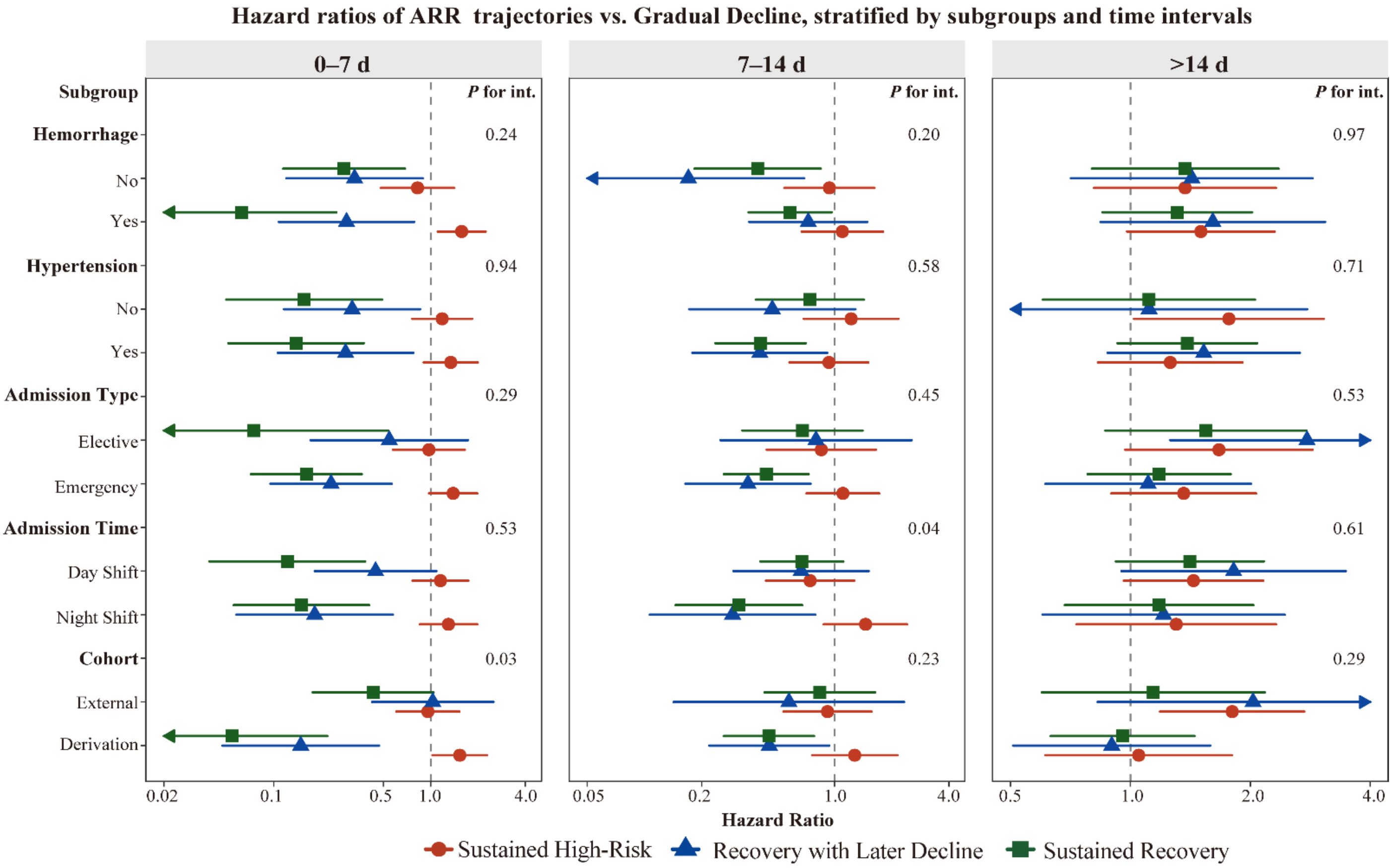

Legend: Forest plots show HRs (95% CIs) from time-stratified multivariable Cox models comparing ARR trajectory phenotypes with Gradual Decline as the reference across additional subgroups. Points denote HRs and horizontal bars denote 95% CIs; the vertical dashed line indicates HR=1. Colors/symbols denote phenotypes: red circles, Sustained High-Risk; blue triangles, Recovery with Later Decline; green squares, Sustained Recovery. "P for int." denotes the likelihood-ratio test for phenotype×subgroup interaction within each time interval.

Supplementary Table 12. Baseline Characteristics of Patients Across Cohorts

Characteristics	Total (n = 8270)	External Validation (n = 3177)	NSICU (n = 5093)	*P*
Age, years	58.9 ± 15.4	61.7 ± 17.4	57.1 ± 13.6	<0.001
Sex, male	4765 (57.6)	1876 (59)	2889 (56.7)	0.037
BMI, kg/m²	25.1 (22.4, 28.4)	27.0 (23.5, 31.7)	24.1 (21.9, 26.4)	<0.001
Admission type	5160 (62.4)	1925 (60.6)	3235 (63.5)	0.008
Admission time				0.006
Day shift	5068 (61.3)	1888 (59.4)	3180 (62.4)	
Night shift	3202 (38.7)	1289 (40.6)	1913 (37.6)	
ICU type	5729 (69.3)	636 (20)	5093 (100)	<0.001
Cerebral infarction	1610 (19.5)	1000 (31.5)	610 (12)	<0.001
TBI	2136 (25.8)	1040 (32.7)	1096 (21.5)	<0.001
Temperature, °C	36.6 ± 0.8	36.6 ± 1.1	36.6 ± 0.6	0.540
Respiratory rate, breaths/min	20.0 ± 9.0	23.5 ± 13.2	17.9 ± 3.4	<0.001
MAP, mmHg	96.9 ± 27.7	93.2 ± 40.6	99.1 ± 14.1	<0.001
Urine output, mL	1980.3 ± 1276.8	2026.6 ± 1784.2	1958.3 ± 945.1	0.043
Heart rate, bpm	88.0 ± 24.3	101.2 ± 30.1	79.8 ± 14.7	<0.001
RBC, $\times 10^{12}$/L	3.8 ± 0.8	3.6 ± 0.9	4.0 ± 0.7	<0.001
Platelet, $\times 10^{9}$/L	185.0 ± 79.4	179.2 ± 90.8	188.6 ± 71.2	<0.001
WBC, $\times 10^{9}$/L	11.5 ± 6.3	13.5 ± 8.7	10.4 ± 4.0	<0.001
BUN, mg/dL	14.5 (10.9, 20.2)	19.0 (13.0, 29.0)	13.2 (10.4, 17.1)	<0.001
ALT, U/L	20.5 (13.9, 34.4)	31.0 (19.0, 62.0)	17.2 (12.5, 26.2)	<0.001
Creatinine, mg/dL	0.7 (0.6, 1.0)	1.0 (0.7, 1.6)	0.7 (0.5, 0.8)	<0.001
Glucose, mg/dL	138.1 ± 67.2	160.3 ± 83.1	124.7 ± 50.9	<0.001
Potassium, mmol/L	3.8 ± 0.5	3.7 ± 0.6	3.9 ± 0.4	<0.001
Sodium, mmol/L	140.4 ± 5.4	139.8 ± 6.2	140.7 ± 4.8	<0.001
INR	1.2 ± 0.7	1.5 ± 1.1	1.0 ± 0.2	<0.001
CKD	1218 (14.7)	351 (11)	867 (17)	<0.001
Hypertension	4814 (58.2)	1663 (52.3)	3151 (61.9)	<0.001
Diabetes	1422 (17.2)	609 (19.2)	813 (16)	<0.001
Liver disease	1363 (16.5)	209 (6.6)	1154 (22.7)	<0.001
CCI	3.0 (2.0, 5.0)	4.0 (2.0, 6.0)	3.0 (2.0, 5.0)	0.009
SOFA	4.0 (2.0, 6.0)	5.0 (3.0, 8.0)	4.0 (1.0, 5.0)	<0.001
GCS at admission	9.9 ± 5.1	10.1 ± 4.5	9.8 ± 5.4	0.006
Ventilation	3512 (42.5)	1497 (47.2)	2015 (39.6)	<0.001
Embolization	2755 (33.3)	295 (9.3)	2460 (48.3)	<0.001
Craniotomy	2578 (31.2)	405 (12.7)	2173 (42.7)	<0.001
Vasopressor use	1250 (15.1)	871 (27.4)	379 (7.4)	<0.001
Dialysis	234 (2.8)	194 (6.1)	40 (0.8)	<0.001
ICP monitoring	1455 (17.6)	473 (14.9)	982 (19.3)	<0.001
Sedation	2736 (33.1)	1091 (34.3)	1645 (32.3)	0.055
Mannitol	1765 (21.3)	189 (5.9)	1576 (30.9)	<0.001
GCS at discharge	11.4 ± 4.2	11.5 ± 4.2	11.4 ± 4.2	0.504

**Discharge location**				<0.001
**Acute/ICU care**	2266 (27.4)	763 (24)	1503 (29.5)	
**Chronic/Long-Term Care**	2310 (27.9)	1270 (40)	1040 (20.4)	
**Home Care/Recovery**	3694 (44.7)	1144 (36)	2550 (50.1)	
**Hospital length of stay, days**	12.3 (8.0, 18.8)	11.8 (6.2, 21.6)	13.0 (9.0, 18.0)	<0.001
**ICU length of stay, days**	7.0 (2.9, 14.2)	6.4 (3.0, 13.0)	7.6 (2.8, 14.6)	0.058
**In-hospital mortality**	1209 (14.6)	750 (23.6)	459 (9)	<0.001

Notes: Continuous variables are reported as mean ± SD or median (IQR), as appropriate; categorical variables as n (%). P values compare NSICU with the pooled external validation cohort (MIMIC-IV and eICU) using the t-test or Wilcoxon rank-sum test for continuous variables and the chi-square or Fisher's exact-test for categorical variables, as appropriate (two-sided).

Supplementary Table 13. CV Performance of Benchmark Machine-Learning Models for In-Hospital Mortality Prediction

Model	AUROC	Accuracy	Brier score	F1 score	CE	PR AUC	Sensitivity	Specificity	NPV	PPV
**AdaBoost**	0.811	0.736	0.128	0.330	0.264	0.338	0.720	0.737	0.964	0.216
**Bayesian Network**	0.822	0.910	0.089	NA	0.090	0.335	0.003	1.000	0.910	NA
**CatBoost**	0.843	0.907	0.068	0.396	0.093	0.419	0.344	0.963	0.936	0.480
**ExtraTrees**	0.854	0.890	0.068	0.416	0.110	0.433	0.435	0.936	0.943	0.407
**GBM**	0.835	0.786	0.098	0.376	0.214	0.367	0.718	0.793	0.966	0.256
**k-NN**	0.788	0.769	0.146	0.326	0.231	0.261	0.621	0.783	0.954	0.223
**LightGBM**	0.839	0.904	0.070	0.369	0.096	0.422	0.316	0.963	0.934	0.457
**Logistic**	0.846	0.718	0.145	0.344	0.282	0.438	0.820	0.709	0.975	0.218
**Naive Bayes**	0.838	0.777	0.178	0.379	0.223	0.377	0.757	0.779	0.970	0.254
**Neural Network**	0.556	0.211	0.229	0.198	0.789	0.125	0.947	0.141	NA	0.115
**Random Forest**	0.858	0.892	0.069	0.438	0.108	0.427	0.470	0.935	0.946	0.417
**CART**	0.741	0.868	0.106	0.298	0.132	0.234	0.309	0.924	0.931	0.320

**SVM**	0.781	0.870	0.091	0.316	0.130	0.287	0.345	0.923	0.934	0.302
**XGBoost**	0.789	0.090	0.171	0.165	0.910	0.300	1.000	0.000	NA	0.090

Note: Values are means across stratified 10-fold CV in the training cohort. AUROC, PR AUC, and Brier score were calculated from predicted probabilities; threshold-dependent metrics were calculated using the fixed F1-optimized threshold of 0.35. CE indicates classification error (1 − Accuracy). NA indicates that the metric was not estimable because the corresponding denominator was zero in one or more folds. Abbreviations: AUROC, area under the receiver operating characteristic curve; PR AUC, area under the precision–recall curve; CART, Classification and Regression Tree; GBM, Gradient Boosting Machine; k-NN, k-Nearest Neighbors; NPV, negative predictive value; PPV, positive predictive value; SVM, Support Vector Machine.

Supplementary Table 14. Performance of Benchmark Classifiers on the Internal Test Set

Model	AUROC	Accuracy	Brier score	F1 score	CE	PR AUC	Sensitivity	Specificity	NPV	PPV
**AdaBoost**	0.807	0.715	0.123	0.324	0.285	0.299	0.762	0.710	0.968	0.206
**Bayesian Network**	0.842	0.911	0.088	0.026	0.089	0.401	0.013	1.000	0.911	1.000
**CatBoost**	0.866	0.914	0.065	0.398	0.086	0.446	0.318	0.973	0.935	0.533
**ExtraTrees**	0.869	0.897	0.066	0.433	0.103	0.423	0.437	0.942	0.944	0.429
**GBM**	0.855	0.798	0.093	0.396	0.202	0.365	0.735	0.804	0.969	0.271
**k-NN**	0.799	0.771	0.143	0.342	0.229	0.269	0.662	0.782	0.959	0.230
**LightGBM**	0.864	0.917	0.066	0.391	0.083	0.450	0.298	0.978	0.934	0.570
**Logistic**	0.871	0.729	0.137	0.364	0.271	0.466	0.861	0.716	0.981	0.231
**Naive Bayes**	0.858	0.793	0.165	0.394	0.206	0.399	0.748	0.798	0.970	0.268
**Neural Network**	0.506	0.090	0.247	0.165	0.910	0.094	1.000	0.000	NA	0.090
**Random Forest**	0.870	0.893	0.067	0.427	0.107	0.411	0.444	0.937	0.945	0.411
**CART**	0.776	0.906	0.086	0.342	0.094	0.304	0.272	0.969	0.931	0.461

**SVM**	0.806	0.884	0.082	0.335	0.116	0.325	0.325	0.939	0.934	0.345
**XGBoost**	0.818	0.090	0.168	0.165	0.910	0.339	1.000	0.000	NA	0.090

Note: Metrics were computed on the internal hold-out set, with threshold-dependent metrics calculated using the fixed F1-optimized threshold.

Supplementary Table 15. Performance of Benchmark Classifiers on the External Validation Set

Model	AUROC	Accuracy	Brier score	F1 score	CE	PR AUC	Sensitivity	Specificity	NPV	PPV
**AdaBoost**	0.682	0.631	0.221	0.455	0.369	0.372	0.652	0.625	0.853	0.349
**Bayesian Network**	0.690	0.755	0.228	0.209	0.245	0.378	0.137	0.946	0.780	0.440
**CatBoost**	0.724	0.717	0.181	0.466	0.283	0.428	0.524	0.776	0.841	0.420
**ExtraTrees**	0.731	0.703	0.161	0.470	0.297	0.429	0.557	0.748	0.845	0.406
**GBM**	0.691	0.652	0.186	0.461	0.348	0.383	0.629	0.659	0.852	0.363
**k-NN**	0.691	0.581	0.294	0.464	0.419	0.361	0.768	0.523	0.879	0.332
**LightGBM**	0.717	0.710	0.190	0.450	0.290	0.411	0.503	0.774	0.834	0.408
**Logistic**	0.716	0.484	0.337	0.454	0.516	0.405	0.907	0.354	0.925	0.302
**Naive Bayes**	0.718	0.553	0.400	0.475	0.447	0.398	0.857	0.459	0.912	0.329
**Neural Network**	0.524	0.236	0.257	0.382	0.764	0.263	1.000	0.000	NA	0.236
**Random Forest**	0.731	0.684	0.163	0.490	0.316	0.424	0.643	0.697	0.863	0.396
**CART**	0.649	0.731	0.224	0.397	0.269	0.359	0.375	0.841	0.813	0.422
**SVM**	0.635	0.728	0.217	0.252	0.272	0.326	0.195	0.892	0.782	0.359
**XGBoost**	0.658	0.236	0.209	0.382	0.764	0.357	1.000	0.000	NA	0.236

Note: Metrics were computed on the external validation cohort, with threshold-dependent metrics calculated using the fixed F1-optimized threshold.

Supplementary Figure 11. Internal Test-Set Performance of the ExtraTrees Classifier

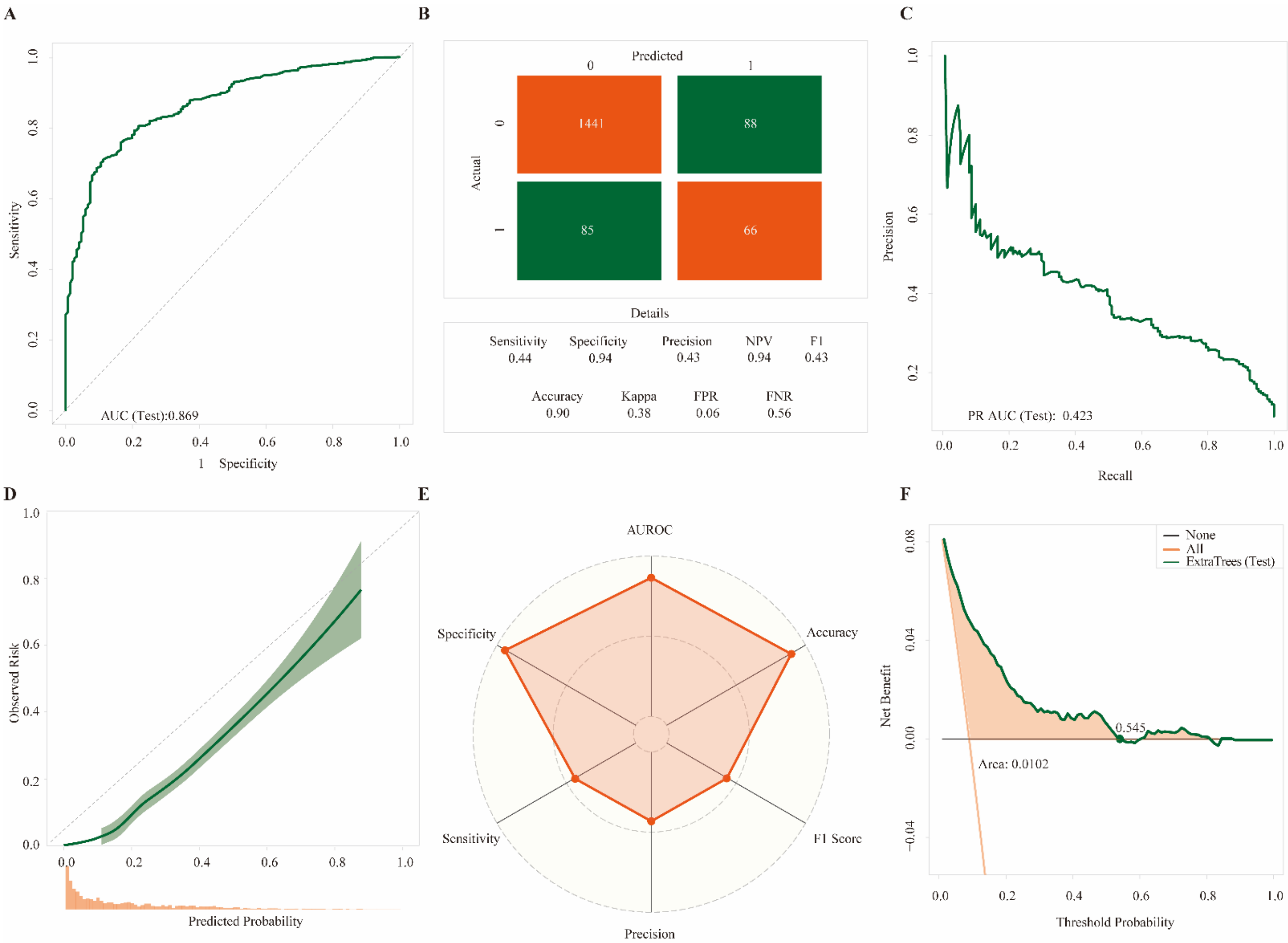

Legend: Panels A–F show the ExtraTrees model evaluated on the internal test set using the prespecified operating threshold from CV. A, receiver operating characteristic curve. B, confusion matrix with accuracy, sensitivity, specificity, precision, F1 score, and Cohen's kappa. C, precision–recall curve. D, calibration plot with a histogram of predicted probabilities. E, radar plot of summary metrics. F, decision-curve analysis with treat-none and treat-all reference strategies.

Supplementary Figure 12. External Validation Performance of the ExtraTrees Classifier

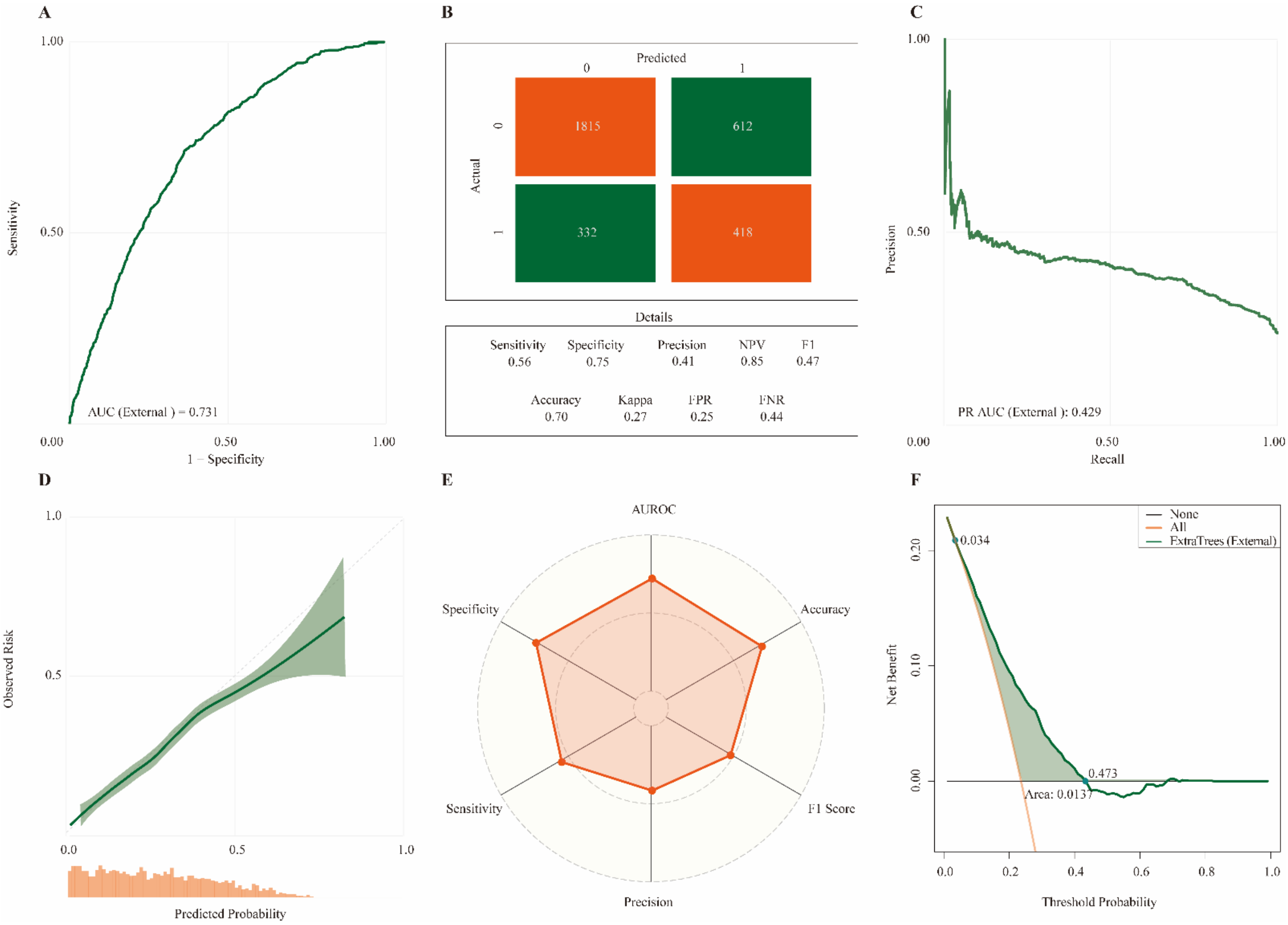

Legend: Panels A–F show the ExtraTrees model evaluated on the external validation cohort using the prespecified operating threshold from CV. A, receiver operating characteristic curve. B, confusion matrix with accuracy, sensitivity, specificity, precision, F1 score, and Cohen's kappa. C, precision–recall curve. D, calibration plot with a histogram of predicted probabilities. E, radar plot of summary metrics. F, decision-curve analysis with treat-none and treat-all reference strategies.

Supplementary Figure 13. Alternative Interpretation of the ExtraTrees Classifier (DALEX & IML)

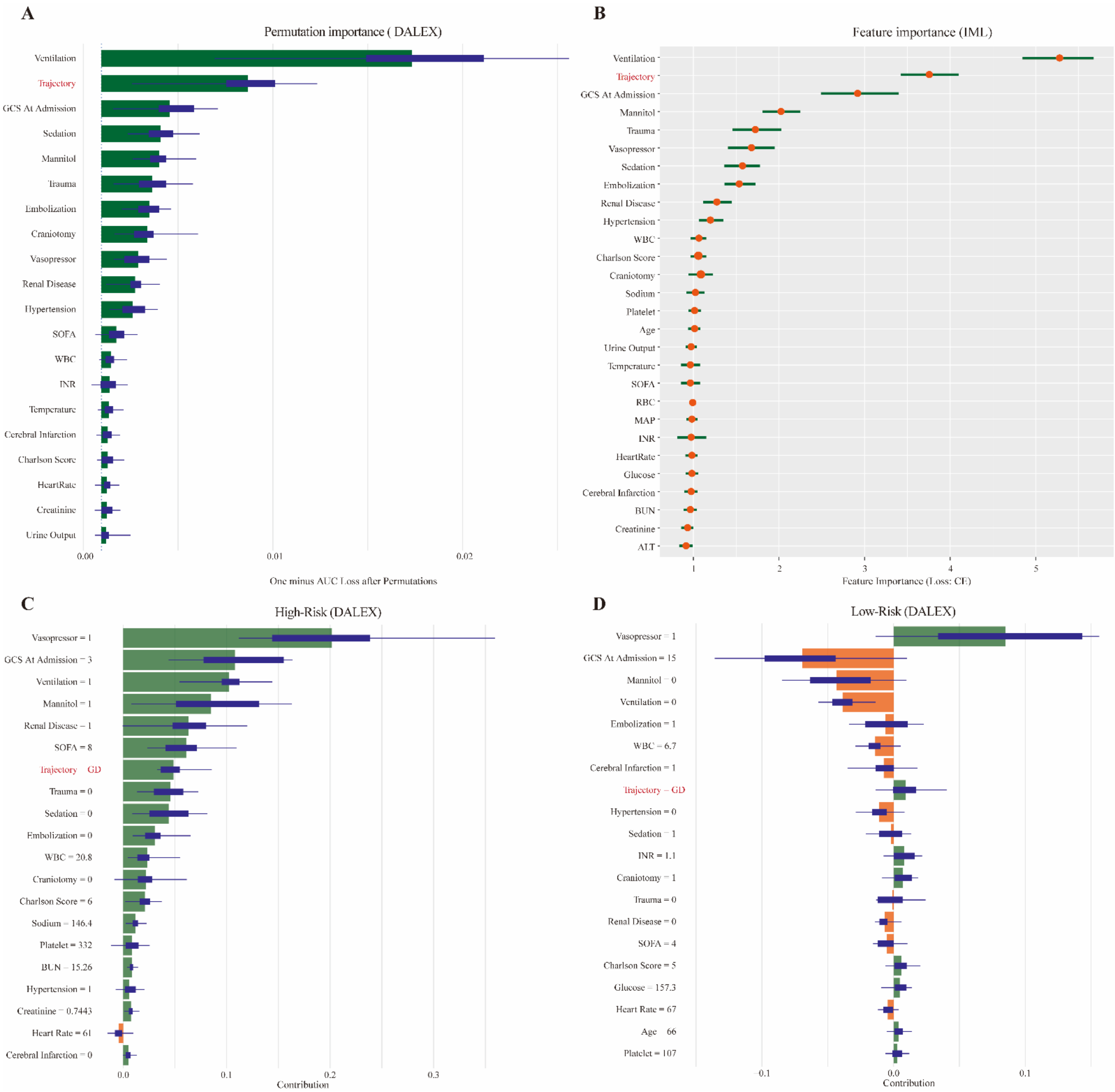

Legend: Panels A–D provide alternative interpretations of the ExtraTrees classifier in the training set. (A) DALEX permutation importance, quantified by the permutation-based change in AUROC for each feature (larger change indicates greater importance; whiskers denote 95% CIs). (B) IML loss-based importance, defined as the increase in classification error after permuting each feature (points with 95% CIs). (C–D) DALEX Break-Down plots for representative high-risk and low-risk patients, decomposing the prediction from the base value (expected model output in the training set) into additive feature contributions; bars to the right increase predicted risk and bars to the left decrease predicted risk.

Supplementary Figure 14. SHAP Dependence Plots for Key Predictors in the ExtraTrees Model

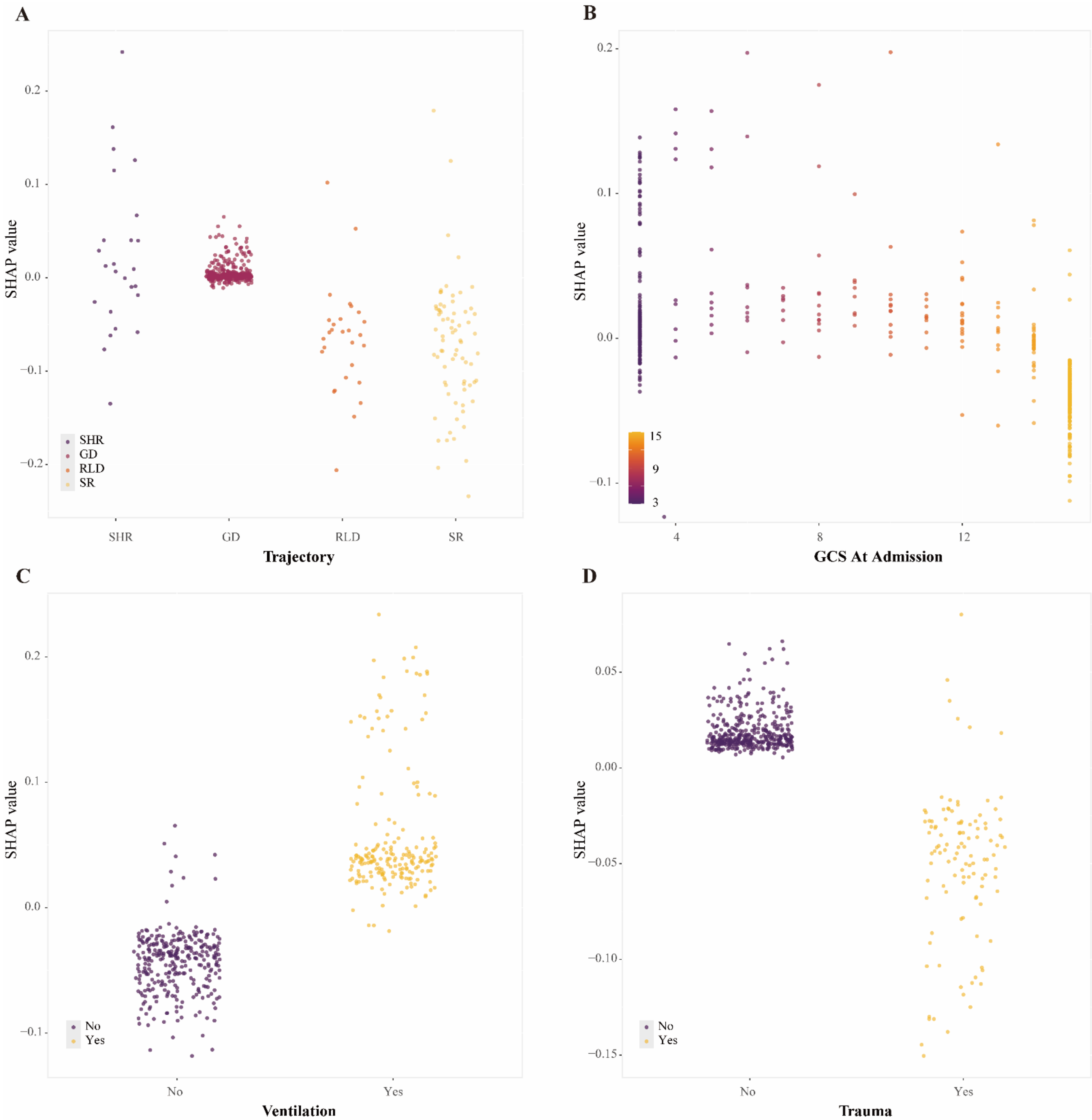

Legend: Panels A–D show SHAP dependence plots in the training cohort (one dot per patient). The x-axis is the feature value and the y-axis is the SHAP value (positive increases, negative decreases the predicted in-hospital mortality risk). Point colors encode feature values (continuous features shown on a low–high gradient; categorical/binary features colored by level). Vertical spread at the same x-value suggests interactions with other predictors.

Supplementary Figure 15. SHAP Dependence Plots Colored by ARR Trajectory Phenotype

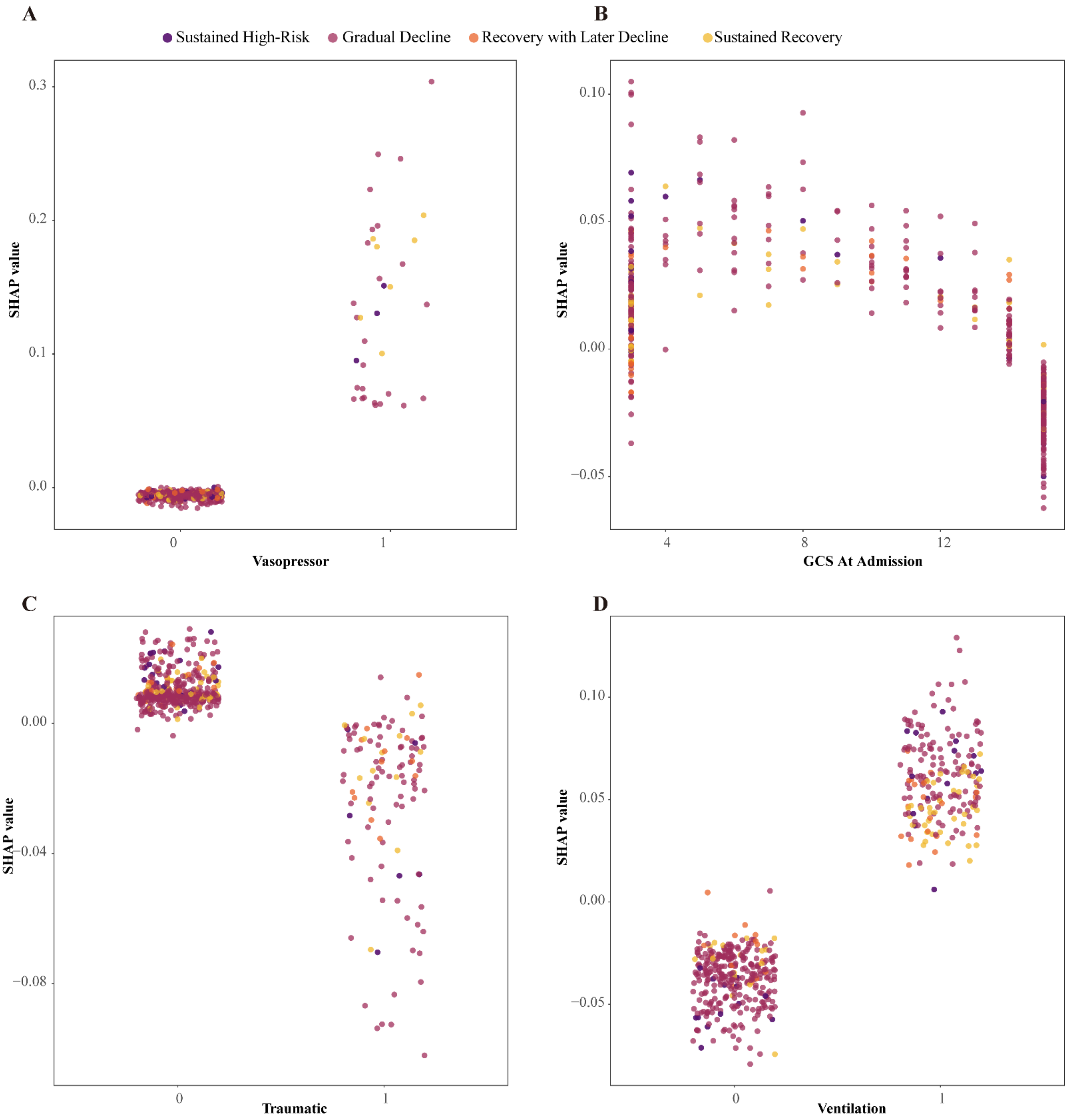

Legend: Panels A–D show SHAP dependence plots in the training cohort (one dot per patient). The x-axis is the feature value (binary variables coded 0/1) and the y-axis is the SHAP value (positive increases and negative decreases the predicted in-hospital mortality risk). Points are colored by ARR trajectory phenotype (Sustained High-Risk, Gradual Decline, Recovery with Later Decline, and Sustained Recovery).

Supplementary Figure 16. Web-Based Prediction Tool for Individualized Risk and Explanation

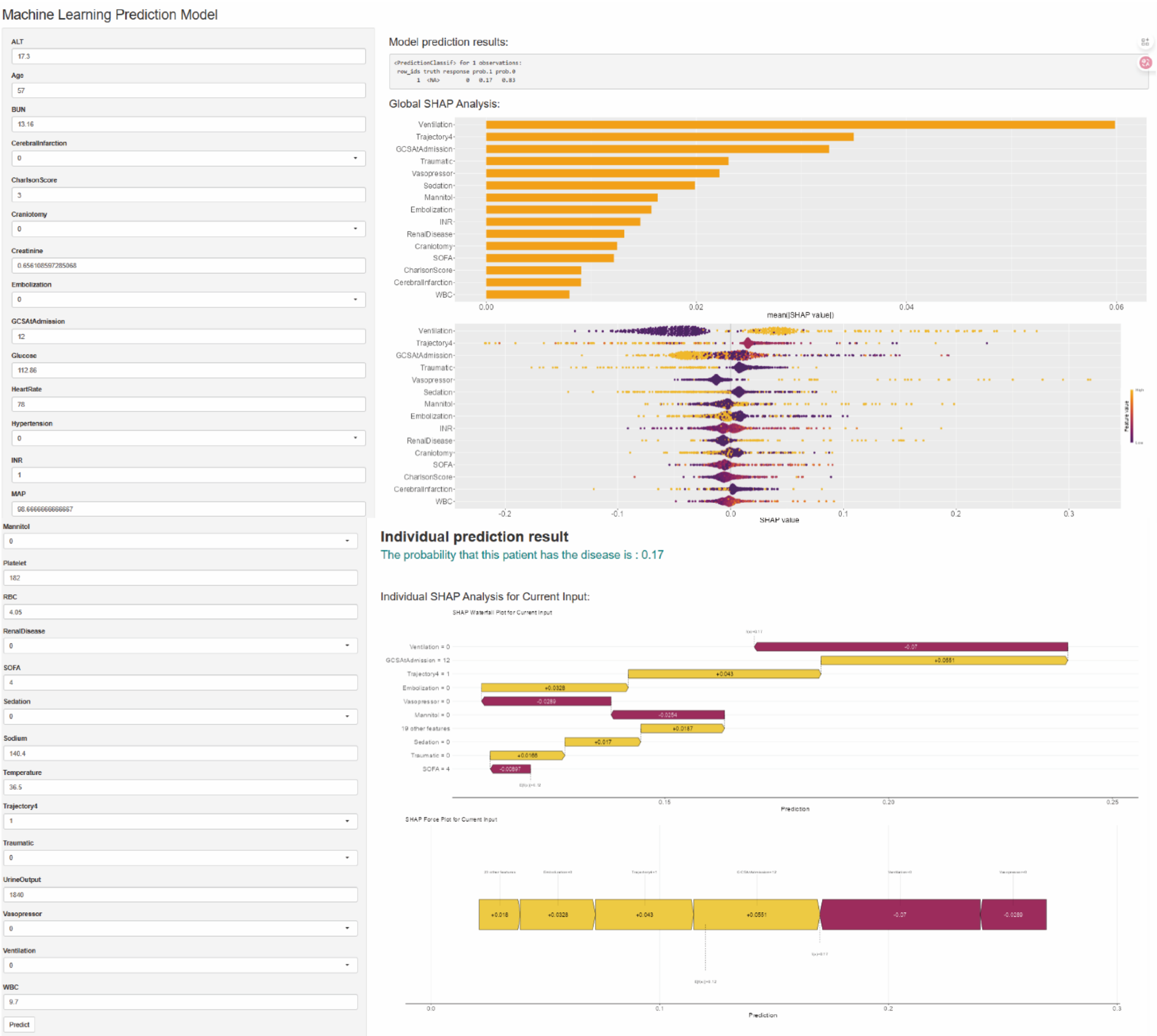

Legend: Screenshot of the public calculator (https://njudrumtowernsicu.shinyapps.io/ABI_Prognosis_Model/) implementing the finalized ExtraTrees classifier. Users enter baseline variables on the left (e.g., ARR trajectory class, GCS at admission, ventilation/vasopressor status, mannitol, SOFA, labs, vitals). The app returns the predicted in-hospital mortality risk (top right) and case-level SHAP explanations: a waterfall plot showing each feature's contribution from the baseline risk to the final prediction (positive = yellow bars, negative = purple bars) and a force plot summarizing the same directionality. The deployed model corresponds to that developed in the training cohort and validated on internal and external cohorts; no patient data are stored by the app. The calculator is provided for research/educational use and should not be used as a sole basis for clinical decisions.
